# HopA1 Effector from *Pseudomonas syringae* pv *syringae* Strain 61 Affects NMD Processes and Elicits Effector-Triggered Immunity

**DOI:** 10.3390/ijms22147440

**Published:** 2021-07-12

**Authors:** Shraddha K. Dahale, Daipayan Ghosh, Kishor D. Ingole, Anup Chugani, Sang Hee Kim, Saikat Bhattacharjee

**Affiliations:** 1 Laboratory of Signal Transduction and Plant Resistance, UNESCO-Regional Centre for Biotechnology (RCB), NCR Biotech Science Cluster, 3rd Milestone, Faridabad-Gurgaon Expressway, Faridabad 121 001, Haryana, India; shraddha@rcb.res.in (S.K.D.); dghosh680@gmail.com (D.G.); kishor.ingole@rcb.res.in (K.D.I.); 2 Kalinga Institute of Industrial Technology (KIIT), Bhubaneswar 751 024, Odisha, India; 3 MedGenome Labs Ltd., 3rd Floor, Narayana Nethralaya Building, Narayana Health City, 258/A, Bommasandra, Hosur Road, Bengaluru 560099, Karnataka, India; anup.c@medgenome.com; 4 Division of Applied Life Science (BK21 Four Program), Plant Molecular Biology and Biotechnology Research Center, Division of Life Science, Gyeongsang National University, 501 Jinju-daero, Jinju 52828, Korea; sangheekim@gnu.ac.kr

**Keywords:** effectors, virulence, PAMP-triggered immunity, effector-triggered immunity, alternative splicing, nonsense-mediated decay

## Abstract

*Pseudomonas syringae*-secreted HopA1 effectors are important determinants in host range expansion and increased pathogenicity. Their recent acquisitions via horizontal gene transfer in several non-pathogenic Pseudomonas strains worldwide have caused alarming increase in their virulence capabilities. In *Arabidopsis thaliana*, *RESISTANCE TO PSEUDOMONAS SYRINGAE 6* (*RPS6*) gene confers effector-triggered immunity (ETI) against HopA1_pss_ derived from *P. syringae* pv. *syringae* strain 61. Surprisingly, a closely related HopA1_pst_ from the tomato pathovar evades immune detection. These responsive differences *in planta* between the two HopA1s represents a unique system to study pathogen adaptation skills and host-jumps. However, molecular understanding of HopA1′s contribution to overall virulence remain undeciphered. Here, we show that immune-suppressive functions of HopA1_pst_ are more potent than HopA1_pss_. In the resistance-compromised *ENHANCED DISEASE SUSCEPTIBILITY 1* (*EDS1*) null-mutant, transcriptomic changes associated with HopA1_pss_-elicited ETI are still induced and carry resemblance to PAMP-triggered immunity (PTI) signatures. Enrichment of HopA1_pss_ interactome identifies proteins with regulatory roles in post-transcriptional and translational processes. With our demonstration here that both HopA1 suppress reporter-gene translations in vitro imply that the above effector-associations with plant target carry inhibitory consequences. Overall, with our results here we unravel possible virulence role(s) of HopA1 in suppressing PTI and provide newer insights into its detection in resistant plants.

## 1. Introduction

Bacterial pathogens are responsible for >10% of the global yield losses in agricultural productivity [1]. Understanding strategies exploited by these rapidly evolving pathogens to evade detection from the host immune system is hence vital for improving food security management approaches. Most bacterial pathogens are genetically equipped with a set of constantly diversifying effector genes that aid not only in physiological adaptations at the host interface but also counter the first layer of immune responses, termed PTI (PAMP-triggered immunity), elicited by the recognition of conserved pathogen-associated molecular patterns (PAMPs) by the plant pattern-recognition receptors (PRRs) [2,3]. Utilizing the Type III secretion system (T3SS), these effectors either suppress key defensive steps of PTI or directly manipulate host metabolic processes to favor pathogen colonization (a process termed as virulence) [4,5,6]. Counter-evolution have endowed more robust surveillance machineries in resistant plants that detect ‘effector-manipulations’ and in turn lead to strong and rapid amplification of defensive measures termed as effector-triggered immunity (ETI). These are orchestrated by specialized class of resistance (R) proteins that bear typical nucleotide-binding (NB) and leucine-rich repeat (LRR) domains. These NB-LRRs are further categorized into CNLs and TNLs depending on coiled-coil (CC) or Toll-interleukin 1-resistance (TIR) domain types present at the N-terminus, respectively [7,8,9]. Effectors that lead to elicitation of ETI are termed as avirulent on the corresponding resistant host. Molecular signatures of ETI share remarkable overlap with PTI and includes processes such as activation of MAPK cascades, production of reactive oxygen species (ROS), callose deposits on cell walls, increased production of defensive hormone salicylic acid (SA), upregulation of defense-associated genes, among others and in selective cases, often in accordance with effector load, is accompanied by localized programmed cell-death called hypersensitive responses (HR) [10,11]. As recently reported, ETI potentiates PTI at a molecular level not only with replenishment of cellular components suppressed by the effectors but also through expression/stability boosts on modulators that drive selective immune signaling routes [10,12,13,14].

*Pseudomonas syringae* (*Ps*) is a major devastating bacterial pathogen with diverse host range worldwide [15]. The *syringae* species is divided into a large number of distinct pathovars based on the plant host from which the strain was originally isolated. Overall, *P. syringae* spp encodes a total of ~57 different Type III effectors (T3Es), although mode of virulence or respective plant target(s) manipulated remain largely unknown for most of these [6,16]. The T3E HopA1 requires a cognate chaperone ShcA for effective translocation and is an important determinant in host range specificity of *Ps* [17,18]. *Ps* pv. *actinidiae* strains isolated from France and Italy was reported to have recently acquired *HopA1* to strengthen virulence on kiwifruit [19]. Similarly, virulence of *P. cichorii*, infecting a wide range of ornamental as well as agricultural crops, is majorly HopA1-dependent [20]. *P. cichorii* with *hopA1^-^* mutation is deficient in biofilm formation and swarming mobility. Together, these reports suggest that HopA1 functions plays important role in virulence of *Pseudomonas* spp. Considering the low level of divergence in HopA1 sequence across different *Ps* pathovars, it is likely that these were acquired through horizontal gene-transfer to expand host range [21].

At a molecular level, HopA1s especially from two different *Ps* pathovars namely *syringae* strain 61 (hereafter named as HopA1_pss_) which infects beans, and pv. *tomato* (*Pst*) strain DC3000 (the corresponding HopA1 hereafter named as HopA1_pst_) remain the two characterized HopA1s till date [22,23]. Sharing 57% amino acid identity and with minor surface charge variations at the structural level, these HopA1s are perceived differently in *Arabidopsis thaliana* Columbia (Col-0), Wassilewskija (Ws-0) or RLD accessions. While HopA1_pss_ triggers ETI in these accessions, HopA1_pst_ does not [22]. RESISTANCE TO PSEUDOMONAS SYRINGAE 6 (RPS6), the cognate R protein that recognizes HopA1_pss_ presence indirectly and activates ETI belong to the TNL class. Activated RPS6 (and other TNLs) connects to immune signaling routes regulated by the central defense modulator ENHANCED DISEASE SUSCEPTIBILITY 1 (EDS1) [24,25]. An *eds1-2* plant is ETI-deficient against HopA1_pss_ [26]. *In planta* interactions of RPS6 with EDS1 and its perturbation by HopA1_pss_ have led to the hypothesis that EDS1 may be a *bona fide* guardee of RPS6 and these disturbances connect a virulence perception to downstream ETI signaling [26]. The mechanistic insight however into how HopA1_pss_ elicits ETI still remains elusive. Independent reports have also implied that putative target(s) of HopA1_pss_ and guardee of *RPS6* is genetically downstream of MAPKs networks transduced by the MEKK1-MKK1/MKK2-MPK4 pathway [27]. However, HopA1_pss_ does not interact with MEKK1, MKK1, MKK2 or MPK4 in yeast two-hybrid assays [28]. Along with RPS6, another TNL SUMM2 also monitors the integrity of MEKK1-MKK1/MKK2-MPK4 network, though *summ2* mutants display wild-type resistance to *PstDC3000* expressing *hopA1_pss_*. Consolidated, clear gaps exist not only in identification of ‘additional’ HopA1_pss_ target(s) but also on the molecular nature of its virulence functions and ETI-elicitation.

Here, we aimed to obtain deeper insights into virulence mode of HopA1s. Using transient expression assays we demonstrate that both HopA1s are capable of eliciting defenses in *Nicotiana tabacum* cv Xanthi and *N. benthamian*a, though the response dynamics differ between the two effectors. Using an inducible HopA1_pss_-expressing transgenic line generated in an *eds1-2* background, we demonstrate that elicitation of ETI and accompanying HR is EDS1-independent. RNAseq analysis on the transgenic line identified considerable similarities to differentially expressed genes (DEGs) of PTI. Notably, upregulation of differential alternative splice (DAS) variants of *TNL*s, which under steady state undergo rapid turnover via the nonsense-mediated decay (NMD) pathway, is especially apparent, overall supporting the recent paradigm that ETI potentiates PTI. However, our data also reveal that in contrast with PTI, DAS *TNL*s are not translated. Enrichment of HopA1_pss_-interactome identifies predominance of protein machineries with known roles in mRNA processing, post-transcriptional/translational regulations and NMD. Interestingly, both HopA1s reduce translational efficiencies of a reporter in cell-free systems. Taken together, our data raises encouraging possibilities that virulence function of HopA1s may involve interference with expression of defense-associated genes at post-transcriptional as well as translational level.

## 2. Results

HopA1 effectors from distinct *P. syringae* pathovars elicit differential strengths of defense responses in plants.

**Figure 1:**
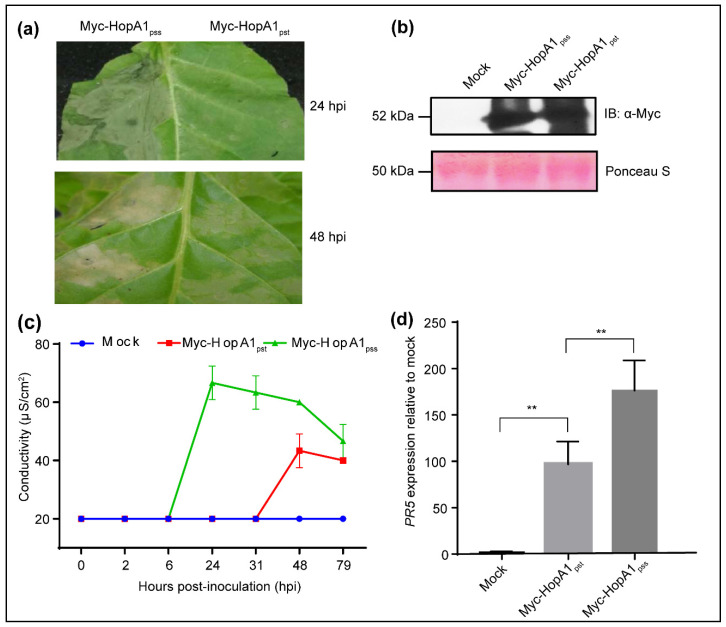
HopA1_pss_ elicits stronger immune response than HopA1_pst_ in *N. tabacum***.** Tobacco leaves were infiltrated either with buffer alone (mock) or with *Agrobacterium* strains expressing Myc-HopA1_pss_ or Myc-HopA1_pst._ (**a**) Hypersensitive response (HR) was imaged at 24- and 48-h post-infiltration (hpi), (**b**) expression levels of Myc-HopA1_pss_ or Myc-HopA1_pst_ proteins determined at 24 hpi via immunoblotting with anti-Myc antibodies, (**c**) electrolyte leakage or conductance assay measured at indicated time points (hpi) and (**d**) expression of *PR5* transcripts determined at 24-hpi. Ponceau S stained membrane shows comparable protein loading between samples. Values are mean ± SD (*n* = 10 for conductance assays, *n* = 3 for qRT-PCRs). Statistical analysis is according to pairwise Student’s *t*-test (** *p* < 0.01).

*Nicotiana* genus is a part of *Solanaceae* family and includes tobacco and tomato. Tobacco is an easy genetic system to use in expression assays via *Agrobacterium*. Transient expression of HopA1_pss_ induces strong HR in *N. tabacum* cv *Xanthi* and *N. benthamiana* [20,29,30]. With 57% amino acid identity to HopA1_pss_, HopA1_pst_ does not elicit HR in *N. benthamiana* [30]. However, when we transiently expressed via *Agrobacterium*, the binary vector clones of Myc-HopA1_pss_ or Myc-HopA1_pst_ in *N. tabacum*, both HopA1s triggered HR (Figure 1a). In *N. benthamiana*, HR-like symptoms were only noted for HopA1_pss_ (Figure S1a). In *N. tabacum*, HopA1_pss_ elicited HR within 24 hpi (hours post-infiltration) while only weak water soaked-like lesions specifically near veins was detected in *N. benthamiana* at 48 hpi (Figure 1a; Figure S1a). Onset of visible HopA1_pst_-induced HR in *N. tabacum* was delayed than HopA1_pss_ and detected at 48 hpi. In *N. benthamiana* HopA1_pst_ remained unresponsive at all time points investigated. Immunoblots showed comparable protein expressions between the two HopA1s across both plant types implying that the degree or timing differences in response are not due to difference in effector expressions (Figure 1b; Figure S1b). To quantify plant responses, we measured the intensity and timing of electrolyte leakage in these responses by performing conductance assays on leaf segments from the infiltrated patch (Figure 1c; Figure S1c). Increasing conductivity by HopA1_pss_ was evident from 6 hpi and plateaued at 24 hpi in *N. tabacum*. In same plants and in accordance with visible symptoms, HopA1_pst_ elicited much delayed conductance increase (initiated at 31 hpi) reaching only half-strength in comparison to HopA1_pss_ at 48 hpi. In *N. benthamiana*, although increased ion leakage was detected at 4 hpi for both HopA1s, similar to *N. tabacum* observations HopA1_pst_ caused lower conductance increments than HopA1_pss_. As defense-associated markers, *PATHOGENESIS-RELATED PR5* or *PR1* expressions were stronger for HopA1_pss_ than HopA1_pst_ in *N. tabacum* and *N. benthamiana*, respectively (Figure 1d; Figure S1d). From these results, it is suggestive that as in Col-0, HopA1_pss_ is sensed more rapidly as a threat and is a stronger immune-elicitor than HopA1_pst_ in either *N. tabacum* or *N. benthamiana*.

### 2.1. HopA1_pss_ Overexpression Elicits ETI in an EDS1-Independent Manner

**Figure 2:**
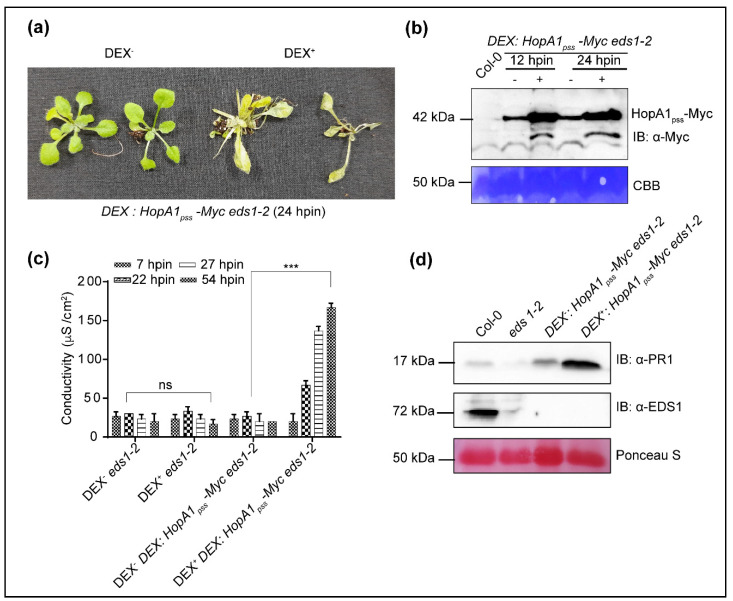
HopA1_pss_ over-expression elicits *EDS1*-independent HR. (**a**) *Dex:HopA1_pss_-Myc eds1-2* transgenic plants without (Dex^-^) and with (Dex^+^) dexamethasone (Dex)-treatment at 24-h post-induction (hpin). (**b**) *HopA1_pss_* protein expressions at 12- or 24 hpin. (**c**) Conductance assay and (**d**) PR1 and EDS1 protein levels in parental (*eds1-2*) and transgenic plants at indicated hpin. Data is representative of mean ± SD (*n* = 3). Pairwise Student’s *t*-test was used to determine statistical significance (*** *p* < 0.001). Loading controls in immunoblots are shown with Ponceau S or Coomassie Brilliant Blue (CBB) staining.

HopA1_pss_ elicits *RPS6*-dependent ETI whereas HopA1_pst_ does not [22,30]. How HopA1_pst_ evades detection and subsequent ETI elicitation is not known. To identify plant targets that may provide more insights into the process, we attempted to generate chemical-inducible [Dexamethasone (Dex)] cMyc-epitope-tagged transgenic lines of each HopA1s in the Col-0 background. Even after repeated attempts, we were unsuccessful. While for HopA1_pss_, obvious ETI elicitation that may occur even with slightest leaky expression may attribute to our failure, the same cannot be explained for HopA1_pst_. As alternate strategies, we selected ETI-deficient *eds1-2* or *rps6-3* plants as the genetic background for generating transgenic plants. Surprisingly, HopA1_pst_ transgenic line in any background was never obtained. Only one transgenic line with HopA1_pss_, named hereafter as *Dex:HopA1_pss_-Myc eds1-2*, was generated in our efforts in *eds1-2* but not in the *rps6-3* background, though both are known to be immuno-compromised to HopA1_pss_ (Figure 2a).

This transgenic line displayed leaky expression of *HopA1_pss_* transcripts, in absence of Dex-application (Dex^−^) that increased ~10-fold at 12 h post-induction (hpin) when sprayed with the chemical inducer (Dex^+^) (Figure S2a). Similarly, HopA1_pss_-Myc protein detected via immunoblotting in Dex^+^ was more elevated than Dex^−^ extracts at 12- or 24-hpin (Figure 2b). The leaky HopA1_pss_ expressions did not affect the growth or seed set properties when compared to either Col-0 or *eds1-2*. However, Dex-treatment (Dex^+^) of *Dex:HopA1_pss_-Myc eds1-2* caused strong HR-like responses at 24 hpin and by 48 hpin the transgenic plants drastically wilted and perished (Figure 2a). These observations suggested that the leaky HopA1_pss_ expressions in Dex^−^ conditions were likely below threshold to cause developmental consequences in the transgenic plants. The HR-like symptoms with Dex^+^ resembled hallmarks of ETI. The occurrence of these in Dex^+^ *Dex:HopA1_pss_-Myc eds1-2* plants hence indicated induction of EDS1-independent ETI responses. Hereafter, we term these responses as ‘ETI^HopA1pss^-like responses’. Electrolyte leakage assays as a measure of ROS production showed progressive increase in conductance in Dex^+^ samples that matched time-dependent increase in wilting (Figure 2c). Electrolyte conductance of Dex^−^ *Dex:HopA1_pss_-Myc eds1-2* plants were similar to *eds1-2* supporting our earlier conclusion that leaky expression of HopA1_pss_ is tolerated in the transgenic plants. Endogenous levels of *PR1* transcripts were ~90-fold higher in Dex^−^ than non-transgenic *eds1-2* control and increased to greater than ~30-fold more at 12 hpin in Dex^+^ extracts (Figure S2b). In accordance with transcript levels, PR1 proteins although marginally elevated in Dex^−^ samples were more prominently detected in Dex^+^ *Dex:HopA1_pss_-Myc eds1-2* extracts. (Figure 2d). As is expected due to *eds1-2*, EDS1 protein was detected only in Col-0 but not in the transgenic plant extracts. Lastly, both Dex^−^ as well as Dex^+^ extracts displayed increased MPK3 and MPK6 phosphorylation, with equal prominence in un-induced and induced transgenic samples, compared to Col-0 or *eds1-2* (Figure S3a). Overall, these results implied that ETI^HopA1pss^-like responses were elicited in the transgenic plants even in the absence of EDS1.

### 2.2. Comparative RNAseq Identifies Events of EDS1-Independent ETI^HopA1pss^-Like Responses

To elucidate molecular features of ETI^HopA1pss^-like responses, we performed RNAseq on total RNA isolated from two biological replicates each from Dex^−^ as well as 12-hpin Dex^+^ *Dex:HopA1_pss_-Myc eds1-2* plants. An average of 40 million paired-end reads were obtained from each sample. Raw reads from each replicate were filtered using Trimmomatic www.usadellab.org (accessed on 3 June 2021) for quality scores and adapters. Filtered reads were aligned to the *Arabidopsis thaliana* (TAIR10) genome using splice aware aligner such as HISAT2 http://daehwankimlab.github.io/hisat2/ (accessed on 3 June 2021) to quantify reads mapped to each transcript. Principal component analysis (PCA) biplot shows low variation across the two biological replicates (Figure S4a). Alignment of reads was in the range of 96% for all samples. RNAseq analysis identified 8726 differentially expressed genes (DEGs), of which 4576 were upregulated and 4150 down-regulated by HopA1_pss_ overexpression and represented in the Volcano plot (Figure S4b,c). Heatmaps for top 50 upregulated and top 10 downregulated genes on a gradient scale based on expression is shown (Figure S4d). Gene ontology (GO) enrichment analyses with ShinyGO software [31] on the basis of biological processes demonstrated that various ‘response to external biotic stimulus’, ‘response to bacterium’, ‘response to stress, populate the up-regulated transcripts while metabolic process-related genes involved in ‘photosynthesis’ and ‘chloroplast organization’ are down-regulated in ETI^HopA1pss^-like responses (Figure S5). The identification especially of defense-associated DEGs support our hypothesis that ETI^HopA1pss^-like responses does not require EDS1.

**Figure 3:**
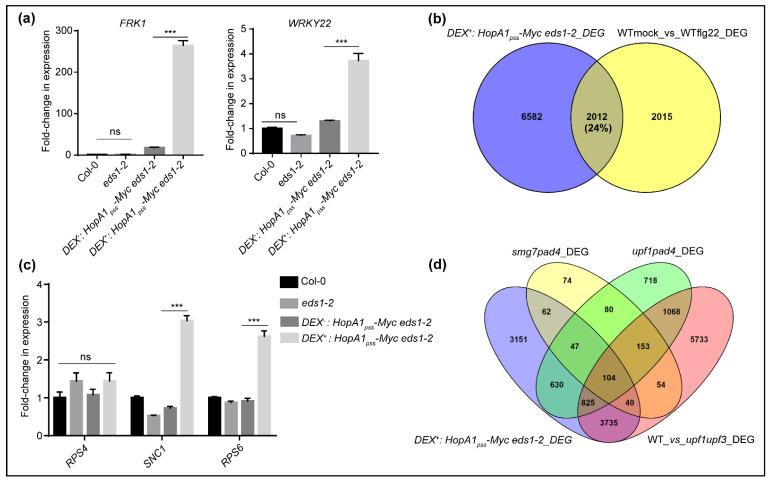
ETI^HopA1pss^-like elicitation upregulates expression of PTI and other defense-associated transcripts. (**a**) Expression levels of PTI markers (*FRK1*, *WRKY22*), and (**c**) *R* genes *RPS4*, *SNC1* and *RPS6* in HopA1_pss_-expressing transgenic at 12 hpin or uninduced control, Col-0 and *eds1-2* plants. Venn diagram of DEG overlaps of Dex^+^ HopA1_pss_ plants with similar datasets from (**b**) flg22-treated versus untreated WT (Col-0) (from Bazin et al., 2020), (**d**) *smg7 pad4*, *upf1 pad4* or *upf1 upf3* versus Col-0 (from Raxwal et al., 2020), Data shown is mean ± SD (*n* = 3) and presented as fold-change relative to Col-0. Statistical significance is with Student’s *t*-test and show pairwise comparisons to Col-0 levels (*** *p* < 0.001).

Recent reports emphasized ETI potentiation of PTI networks [13,14,32]. In agreement to this, our RNAseq data contained upregulated expression of a vast majority of PTI-responsive genes such as *BAK1*, *SOBIR1*, *BIK1*, *MPK6*, *NHL10*, *FOX1*, *WRKY29*, *FRK1*, *MKK4*, *MKK5,* among others in Dex^+^ than Dex^−^ *Dex:HopA1_pss_-Myc eds1-2* samples. Increased expression of selected PTI markers such as *FRK1*, *WRKY22*, *WRKY29*, *WAK2*, *FOX* and *CYP81F2* were tested and validated by qRT-PCRs analysis (Figure 3a, Figure S6). We also noted upregulated expression of *PAD4* and SA-biosynthetic enzyme *ICS1* in the Dex^+^ *Dex:HopA1_pss_-Myc eds1-2* samples. When compared to Col-0 or *eds1-2*, a modest upregulation for some of the above transcripts (such as *FRK1*, *WAK2*, *FOX*, *WRKY29* and *ICS1*) was noted in the Dex^−^ samples, possibly due to the leaky expression of HopA1_pss_ in the transgenic plants. Nevertheless, Dex^+^ samples had more elevated levels of these transcripts. It is interesting to mention here that while *BIK1* and *MPK3* upregulations in ETI^AvrRps4^ is EDS1-dependent [13], as our RNAseq data indicated for ETI^HopA1pss^-like responses, it is clearly not. Whether these observations implicate EDS1-requirement in AvrRps4-, but not HopA1_pss_-perception for ETI-elicitation thus hinting on cellular locales/processes where the corresponding avirulent effectors are intercepted remains an encouraging possibility to consider. To determine the proportion of PTI DEGs represented in our data, we compared these to the DEG from flg22-treated Col-0 [33]. In the ~24% overlap we detected with our DEGs, several NLRs, lectin-receptors such as kinase, *MPK11*, *MKK4*, splicing factors such as *SR45* and different ribosomal proteins such as *RPL10B*, phospholipase D, different F-box protein coding genes and WRKY transcription factors were identified (Figure 3b). This overall low DEG overlaps may be because of lack of EDS1, a known player in PTI, in our transgenic plants.

### 2.3. ETI^HopA1pss^-Like Responses Possess Hallmarks of PTI Potentiation

HopA1_pss_–mediated ETI signaling by activated RPS6 requires EDS1 [22,26]. Even though *RPS6* did not feature in our DEGs, steady state transcript levels of ~30 TNLs including *RPP4*, *SNC1*, *RML1A*, *RPP1*, *CHS1* were higher in our Dex^+^ DEG list (Table S1). We performed qRT-PCRs on the same samples as was submitted for RNAseq and detected strong increase in *RPS6* and *SNC1* but not *RPS4* transcript levels (Figure 3c). TNL expressions are regulated post-transcriptionally by alternative splicing (AS) and utilize the NMD pathway for turnover to prevent their mis-primed activations [34]. Features of long 3′-UTR along with numerous introns make the *TNL* transcripts ideal targets for NMD. The RNA helicase Up-Frameshift 1 (UPF1), along with its accessory proteins UPF2, UPF3 and the phosphoserine binding protein SMG7 recruits deadenylation, decapping and RNA exo- and endonucleases to execute the NMD of *TNL* transcripts [35]). In NMD-deficient mutants such as *upf1-5*, *upf3-1* or *smg7-1 TNL* expressions are upregulated conferring enhanced resistance to *PstDC3000* infections [36,37,38]. PTI triggered by *PstDC3000* also causes 26S proteasome-mediated progressive degradation of UPF proteins at fairly early time points that relieve NMD suppressions [39,40]. Further, auto-immunity in *smg7* plants is *RPS6*-dependent though NMD-deficiencies remain *EDS1*- or *SID2*-independent [39]. Not the least, *upf1-5* and *smg7* contribute to differential degree of NMD suppressions [41].

Thus, with indications of NMD perturbations apparent by the detection of upregulated *TNL*s in ETI^HopA1pss^-like responses, we compared the percentage of DEG overlaps to those recently reported for *smg7 pad4*, *upf1 pad4* or *upf1 upf3* plants [39,41]. We reasoned that lack of *EDS1* in our Dex^+^ *Dex:HopA1_pss_-Myc eds1-2* system is immuno-consequentially similar to the absence of *PAD4* in *smg7 pad4* or *upf1 pad4* and hence may allow for more accurate comparisons. Growth abnormalities of *smg7* is completely abolished by *pad4-1*, however it does not rescue the same for *upf1*. Interestingly, NMD perturbations in either *smg7* or *upf1-5* is *PAD4*-independent. Taken together with our observation that Dex^+^ *Dex:HopA1_pss_-Myc eds1-2* plants show HR hence implied that analogous to *smg7* or *upf1-5*, NMD interference likely will remain unaffected. Whereas only insignificant (~3%) similarity with *smg7 pad4* was noted, DEG overlaps were in the range of 18% or 55% with *upf1 pad4* or *upf1 upf3* list, respectively (Figure 3d). These results therefore hinted that DEGs in ETI^HopA1pss^-like responses originated from disturbances in UPF-, but not SMG7-mediated effects on NMD pathway.

MPK3/6 but not MPK4 directs UPF instabilities during PTI [40], To elucidate whether the observed NMD alterations in the ETI^HopA1pss^-like responses are channeled through the activated MAPKs signaling routes, we then compared DEG overlaps to PTI-induced (flg22-treated) data from *mpk3*, *mpk4* or *mpk6* mutants reported recently [33]. DEG overlaps with the *mpk3* (flg22) or *mpk6* (flg22) were dramatically low (4% or 2.5%, respectively) whereas a moderate increase (34%) was noted with *mpk4* (flg22) datasets (Figure S3b). Therefore, taking into account our earlier result of MPK3/6 activation in *Dex:HopA1_pss_-Myc eds1-2* extracts, these results implied that ETI^HopA1pss^-like responses recruited MPK3/6 networks in NMD suppressions and PTI potentiation.

### 2.4. ETI^HopA1pss^-Like Responses ‘Short Circuits’ NMD Suppressions Downstream of MPK4

**Figure 4:**
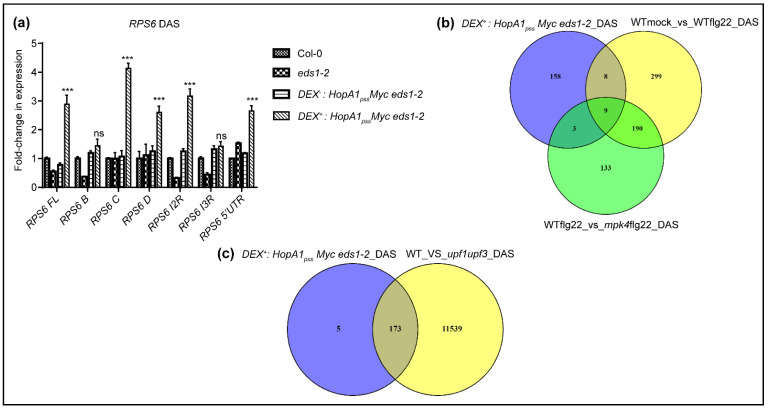
Alternative splicing of transcripts are perturbed during ETI^HopA1pss^-like responses. (**a**) Expression level of splice variants of *RPS6* in Col-0, *eds1-2*, Dex^−^ and Dex^+^ HopA1_pss_-expressing transgenic plants. Data represent mean values from triplicate samples (± SD) and are indicated as fold-change relative to Col-0. Pairwise Student’s *t*-test was used for statistical analysis (*** *p* < 0.001). Venn diagram showing overlap between unique differentially alternatively spliced (DAS) transcripts in early ETI^HopA1pss^ to (**b**) flg22-treated WT (Col-0) or *mpk4* plants (from Bazin et al., 2020) and (**c**) *upf1 upf3* mutant (from Dreshel et al., 2013).

During PTI, MPK4- but not MPK3/6-signaling networks are instrumental in transducing flg22-induced alternative splicing (AS) events that cause increase in steady-state transcript levels of splice variants especially of *TNL*s [33]. Indeed, several splicing factors orchestrating AS are direct phosphorylation targets of MPK4 [42]. DAS (differentially alternatively spliced) transcripts with roles in mRNA processing and splicing functions are also markedly enriched in *upf1 pad4* DEG data implying repercussions on AS due to NMD perturbations [41]. To evaluate whether NMD defects in ETI^HopA1pss^-like responses affect AS, we first determined the steady-state levels of *RPS6* splice variants in Dex^+^ versus Dex^−^ samples via qRT-PCRs. Indeed, several *RPS6* AS variants were upregulated in the Dex^+^ samples [39] (Figure 4a). This result further instigated us to comprehensively mine our transcriptome data for changes in DAS events. Using the rMATS software [43], a total of 229 DAS events were identified in ETI^HopA1pss^-like responses when compared to the control (Dex^−^) samples and mapped to 178 unique DAS genes (Table S2). GO enrichments categorized these to diverse defense-associated, signal transduction, mRNA processing and cell-to-cell communication related processes. Remarkably, both positive and negative immune regulators such as *GRP3* (Glycine-rich protein 3), *ATG2* (Autophagy-related 2), *CC1* (Classification Criterion 1), *CPK28* (Calcium-dependent protein kinase 28), *GAT1* (GABA Transporter 1), *LIF2* (LHP1-interacting factor 2) and others were present in the DAS list.

As maybe anticipated, these DAS displayed less than 1% overlap to the DEGs, in agreement with their lower representations noted in most transcriptome data [33,44] (Figure S7). To the 546 PAMP-induced DAS events (mapping to 506 unique genes) identified by Bazin et al. (2020), we detected only a minimal overlap (~10%), again possibly due to lack of immune amplifications by EDS1 (Figure 4b). Nevertheless, at least three splicing factors *SR30*, *SR34*, *RZ1C* and several other candidates such as *CPK28* (Calcium-dependent protein kinase 28), *LSD1* (Lesion-simulating disease 1), were shared with the PTI-induced DAS list [33]. Similarly, less than 7% overlap was also observed between our DAS list to those reported for AS-disturbed *mpk4* mutant [33] (Figure 4b). Further classification of ETI^HopA1pss^-like associated DAS events revealed 31% 3′-alternative splicing (3ASS), 25% retention (IR), 21% exon skipping (SE), 19% alternative 5′-splicing (5ASS) and 2% mutually exclusive exons (MXE) events (Figure S8). As was evident, the categorizations were different from flg22-induced DAS reported in either Col-0 or *mpk4* plants [33]. These comparisons implied that AS alterations in ETI^HopA1pss^-like responses are orchestrated distinct to PTI and recruits signaling routes downstream of MPK4, as genetically predicted in earlier studies [27,28,39]. To determine whether DAS transcripts identified in these responses are indeed NMD targets accumulated due to UPF-perturbations, we compared them to DAS dataset reported for *upf1 upf3* plants. These double mutants are impaired in AS-coupled NMD [45]. Remarkably, 173 of 178 DAS transcripts of ETI^HopA1pss^-like responses were present in the *upf1 upf3* list (Figure 4c). DAS representations of selective genes (*CPK28*, *SNC1*, *SR30*, *SR34*, *LSD1* and *GRP3*) are shown (Figure S9). From these analyses, we speculate that while PTI utilizes extensive MAPK-cascade phosphorylation events to cause UPF turnover and modulate functions of several splicing-associated proteins [46,47], ETI^HopA1pss^-like responses ‘intersect’ on selective nodes (MPK3/6, but not MPK4) of this network for NMD-suppressions. Whether this indeed is individual for ETI^HopA1pss^ and not the other ETI system remains to be determined further.

### 2.5. HopA1_pss_ Interactome in Planta Is Enriched for Multiple RNA Process-Associated Proteins

**Figure 5:**
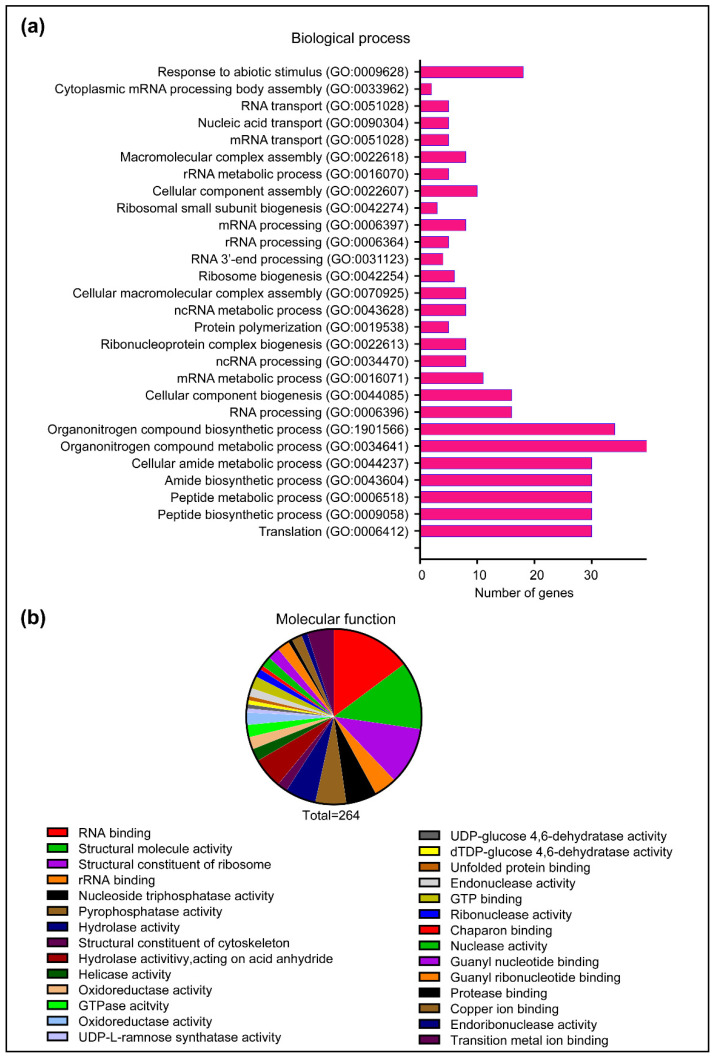
Proteins associated with RNA-related processes are enriched with HopA1_pss_. Gene ontology (GO) categorization of proteins co-eluted with HopA1_pss_ immuno-enrichments from plant samples, in terms of (**a**) biological processes or (**b**) molecular functions.

To identify plant targets of HopA1_pss_ and obtain deeper insights into events that lead to ETI^HopA1pss^, we harvested tissues from Dex^+^ *Dex:HopA1_pss_-Myc eds1-2* plants (at 12 hpin) or leaves from *N. benthamiana* leaves transiently expressing Myc-HopA1_pss_ (at 48 hpi) (Figure S10). Immuno-enrichments on independent biological replicates from each system were performed with anti-Myc antibody conjugated agarose beads or with IgG-conjugated beads as non-specific binding controls. Bound proteins were digested with trypsin, the resulting peptides were subjected to LC-MS/MS, and their identities determined by database searches. A total of 207 *Arabidopsis* and 59 *N. benthamiana* unique proteins that co-eluted with HopA1_pss_ were identified across different sample replicates (Tables S3 and S4). A considerable overlap in HopA1_pss_ co-eluted proteins was noted between the enrichments from these two distinct plant systems thus providing reasonable endorsement to their accuracies (Table S5). Gene ontology (GO) analysis categorized the HopA1_pss_-interactome to 28 GO terms by ShinyGO v0.61: Gene ontology enrichment analysis http://bioinformatics.dstate.edu/go/ (accessed on 10 June 2021) [31] encompassing diverse biological processes (Figure 5a). With GO enrichments and classification according to molecular functions, a predominant presence of proteins associated with RNA-binding processes was especially apparent (Figure 5b). These included Decapping protein 5 (DCP5), Polyadenylate-binding proteins (PABP2/4/8), THO complex protein 4B (ALY2), Glycine rich RNA-binding proteins (GRP7, GRP8), DEAD-box ATP-dependent RNA helicases (RH3, RH52) as well as different ribosomal proteins of 40S and 60S subunits, among others. In the protein-protein interaction (PPI) network (STRING database with high confidence), functional interconnections between the identified candidates are evident (Figure S11). These associations may hold vital clues to cellular process/locale of HopA1_pss_ virulence and perception in resistant plants.

Most importantly, we noted that in support with our earlier speculation regarding ETI^HopA1pss^ may initiate from UPF-associated NMD disturbances, several HopA1_pss_-interacting proteins such as GRP7, GRP8 and PABPs indeed have previously reported activities that influence AS [48,49]. Additionally, PABPs and RNA helicases (e.g., RH8) are known interacting partners of UPF1, and DCP5 co-purifies with it [35]. Taken in context of UPF1 recruitment deficiencies in *smg7* that cause mis-primed activation of RPS6 [39], the HopA1_pss_-interactome we identified may hence hold promising clues to explore its virulence functions and ETI elicitation in resistant accessions.

### 2.6. HopA1_pss_ Suppresses Reporter Gene Translations In Vitro

**Figure 6:**
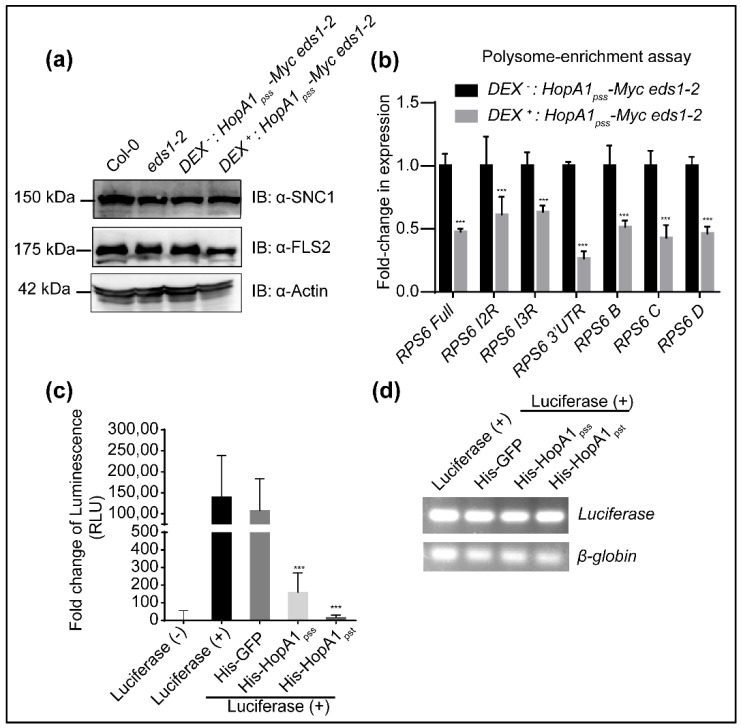
HopA1s display translational suppression activity. (**a**) Expression levels of SNC1 and FLS2 proteins in Col-0, *eds1-2*, Dex^−^ and Dex^+^ HopA1_pss_-expressing transgenic plants at 12 hpin. Immunoblots were performed with anti-SNC1, anti-FLS2 or with anti-actin as representative of loading control. (**b**) Transcript levels of *RPS6* AS variants enriched on polysomes during ETI^HopA1pss^-like response. Values are mean ± SD of three replicates and shown as relative to Dex^−^ levels. Statistical analysis is with pairwise comparison to Dex^−^ or Luciferase (-) samples by Student’s *t*-test (*** *p* < 0.001). (**c**) HopA1_pss_ and HopA1_pst_ suppress reporter gene expression in in vitro transcription-translation coupled assay system. *Luciferase* (*LUC*) activity in the presence of equimolar amounts of His-HopA1_pss_, His-HopA1_pst_ or His-GFP (negative control). (**d**) Semi-quantitative qRT-PCR of *LUC* and control *β-globin* expression in the above samples.

A previous search of structural homologs of HopA1_pst_ identified remote similarities with eukaryotic translation initiation factor 4E (eIF4E) [23]. A search for structurally similar proteins to HopA1s (HopA1_pss_ PDB code: 4RSW; HopA1_pst_ PDB code: 4RSX) using the DALI server http://ekhidna2.biocenter.helsinki.fi/dali (accessed on 6 June 2021) showed highest similarity to eIF4E (Table S6). Even though eIF4E *per se* was not identified in HopA1_pss_ enrichments, presence of GRP7/8, PABPs, DCP5 and others with known translational roles and as eIF4E-interactors taken together with UPF1 involvement in translational suppression of NMD-targeted mRNAs that also included *TNL*s, hints at possible HopA1_pss_ interference on these processes [41,50,51]. To explore this, we performed anti-SNC1 immunoblot on Dex^−^ or Dex^+^ *Dex:HopA1_pss_-Myc eds1-2* extracts (with Col-0 or *eds1-2* as controls). We noted that SNC1 protein levels remained comparable between these samples suggesting that enhanced *SNC1* transcripts although upregulated in ETI^HopA1pss^-like responses do not undergo increased translation (Figure 6a). To validate this phenomenon, we extended our investigations to determine translation efficiencies of *RPS6* AS splice variants, several of which we noted earlier were upregulated in ETI^HopA1pss^-like samples. Since anti-RPS6 antibodies were not available, we compared polysome associations of *RPS6* splice variants between Dex^−^ or Dex^+^ *Dex:HopA1_pss_-Myc eds1-2* samples. Remarkably, induction of ETI^HopA1pss^-like responses resulted in significant reduction in polysome enrichments of *RPS6* splice variants compared to Dex^−^ samples (Figure 6b). Clearly, global translation was not affected since neither Actin nor FLS2 protein accumulations varied between the different samples (Figure 6a). Interestingly, neither *Actin* nor *FLS2* are NMD targets. Overall, these results seem to suggest that HopA1_pss_ virulence properties likely orchestrate post-transcriptional or translational suppression of defense-associated transcripts including *TNL*s that are activated by PTI-mediated turnover of UPF proteins and its effect on NMD processes.

To estimate effects on overall translation efficiencies we utilized the rabbit reticulocyte lysate-driven in vitro coupled transcription and translation assay to compare the activity of Luciferase (LUC) reporter synthesized in presence or absence of purified recombinant HopA1_pss_. Additionally, whether comparable or differential effects are noted thus accounting for its differential response *in planta*, we also included recombinant HopA1_pst_ in these assays. Most NMD machineries are conserved between plant and animal systems [52,53]. Equimolar amounts of the GFP protein were used as a negative control in these reactions. Remarkably, presence of either HopA1_pss_ or HopA1_pst_, but not GFP, strongly suppressed LUC activity (Figure 6c). Heat-denatured HopA1_pss_ or HopA1_pst_ was deficient in this suppression indicating a strict requirement of their native conformations for translational interference functions (Figure S12). In semi-quantitative PCR assays, transcription efficiencies of *LUC* reporter remained unaffected by the presence of either HopA1 and were comparable to the GFP-control (Figure 6d). Thus, both HopA1s possessed the propensity to suppress gene expressions post-transcriptionally. In summary, our investigations here present events of early ETI^HopA1pss^ that potentiates PTI at specific nodes and also identifies putative targets/processes that HopA1 effectors may attempt to manipulate as its virulence function.

## 3. Discussion

Pathogen-encoded effectors are strong determinants of success rates in pathogenesis. Not only they affect the pathogen physio-biology, but also are pivotal in determining aggressive expansion of host range [3]. Under a constant threat of detection by the plant surveillance system these effector repertoires undergo continuous evolutionary diversification retaining mutations that overall benefit in pathogen survival [21]. The HopA1 class of conserved effectors in *Pseudomonas* pathovars and its differential perception in plants provides an excellent system to study reciprocal aspects of host and invader adaptations in plant-pathogen interactions. HopA1_pss_ elicits ETI in Col-0 but HopA1_pst_ does not [17,22,54]. Our studies here identify responsive difference between the two HopA1s also in *N. tabacum* and *N. benthamiana*. Kang et al. (2021) recently reported HR elicited by transient expression of HopA1_pss_ in *N. benthamiana*. HopA1_pst_ expressed leaves remained asymptomatic. Overall, our results are consistent with their study and even though visibly *N. benthamiana* appears unresponsive to HopA1_pst_, defenses at much weaker intensities than HopA1_pss_ are indeed elicited as revealed by progressive increase in conductance and enhanced expression of *PR1*. These results suggest that similar to Arabidopsis, both *N. tabacum* and *N. benthamiana* likely retain conserved immune sentinels for HopA1_pss_ perception and trigger of immunity [22,26]. Similarities in HopA1_pss_-interacting proteins we enrich from *Arabidopsis* or *N. benthamiana* provides rationale to our hypothesis. In addition, weaker immune responses detected to HopA1_pst_ also lead us to speculate that it may have evolutionarily acquired sequence divergence from HopA1_pss_ to evade detection in planta. Interestingly, in structural studies differences in surface electrostatic charge between the two HopA1s are noted, and it remains to be investigated further with mutational and swapping approaches whether the attributing residues are responsible for their differential perception in plants [23].

We were extremely surprised that our success was limited to obtaining a single Dex-inducible HopA1_pss_ transgenic line in an *eds1-2*, but never in Col-0 or *rps6* background. While this warrants more efforts, Kang et al. (2021) recently reported generation of multiple Estradiol-inducible HopA1_pss_-expressing transgenic plants in the RLD accession, implying that genetic background for one may be an important contributing factor. The RLD accession though encodes RPS6, is known to lack functional RPS4 and SNC1 TNLs [22,55]. Developmental penalties in plants with constitutive activation of SNC1 is well documented [56]. Therefore, with unregulated TNL activations observed in ETI^HopA1pss^-like responses, lethality may not be surprising in a Col-0 background. Mild growth deficiencies and increased accumulation of defense-associated proteins are also noted in the RLD transgenic with leaky expression of HopA1_pss_ [30]. Genetically, *eds1-2* mutation is expected to mitigate TNL-mediated defenses and EDS1 is required for transcriptional upregulation of *PR1* during ETI [24,57]. However, increased accumulation of *PR1* transcript and protein together with the HR triggered in our transgenic plants upon Dex-treatment implies that ETI^HopA1pss^ elicitation is either downstream of EDS1 or defenses due to over-expressed HopA1_pss_ may bypass the requirement for EDS1.

In addition to the above, our failure in generating inducible HopA1_pst_-expressing transgenic plants in any of the genetic background we used here also remains an enigma. We conceive two possible explanations for this. Firstly, in the absence of a counter response from plants (such as elicited ETI for HopA1_pss_) that attempts to mitigate effector activities, plant survival is compromised by unregulated HopA1_pst_ expression and its virulence activities. Secondly, lack therein of other effector armory as would be co-secreted in a physiological invasion may also be the cause for lethality in HopA1_pst_-expressing plants. Effector–effector interactions (termed as meta-effector effects), regulations in context of expression levels and translocation efficiencies collectively are important determinants in disease outcomes on a given host [58]. Several *PstDC3000* effectors are known to suppress ETI including those elicited by HopA1_pss_ implying that either same, related or components of connected defense signaling routes are targeted by at least more than one effector [59]. In *Legionella pneumophila*, effectors influencing either the stage-dependent stability of other effectors or antagonizing their actions have been reported [58]. Thus, in the absence of other intersecting effector functions HopA1_pst_ virulence is not fine-tuned and its overshoot likely led to lethal consequences on the expressing plants. Our observations that both HopA1s possess comparable translational suppression activity in vitro presents a possibility that their targets in Arabidopsis include vital survival-associated transcripts. Comparative RNAseq of polysome associated transcripts between Dex^−^ and Dex^+^ samples of *Dex:HopA1_pss_-Myc eds1-2* transgenic plants will provide more insights into this hypothesis.

Our RNAseq data on ETI^HopA1pss^ provides several novel insights. Foremost, DEGs identified in the ETI^HopA1pss^ samples unequivocally support recent demonstration of ETI potentiation on PTI responses [13,14]. An inducible ETI^HopA1pss^ system was not included in previous studies. We detected significant upregulations of several PTI markers in our ETI^HopA1pss^-like datasets even though overall DEG overlaps with reported PAMP-DEGs [33] was in the lower quarter percentile, perhaps due to lack of EDS1 role in PTI [60,61]. Nevertheless, upregulated expression of *BIK1* or *MPK3* and increased MPK3/6 phosphorylation which are EDS1-dependent in ETI^AvrRps4^, contrastingly was EDS1-independent for ETI^HopA1pss^ [12]. These results lead us to surmise that connecting modes to PTI enhancements from ETI may be distinct and individualized according to the perception of the cognate avirulent effector. Secondly, significant upregulation of multiple TNLs that also occur in the *upf1* mutant (such as *RPS6*, *SNC1, SOC3, RPP13, At5g38340*) are noted in our dataset thereby supporting that ETI^HopA1pss^ displays AS-NMD suppression similar to UPF functional inhibitions in PTI [40,41]. We were unable to check levels of UPF proteins in our ETI^HopA1pss^-like extracts due to lack of appropriate antibodies. Nevertheless, 97% overlap in our early ETI^HopA1pss^ DAS candidates with DAS from *upf1 upf3* support that UPF functions are likely suppressed.

Crosstalk between AS and NMD is essential for regulating gene expressions especially during immunity [62,63]. Interestingly, identified DAS in ETI^HopA1pss^-like responses includes both positive and negative immune regulators. GRP3 is a negative regulator of immunity and prevents activation of Wall-Associated Kinase 1 (WAK1), a receptor-like kinase (RLK) for the DAMP (Damage-associated molecular patterns) oligogalaturonides (OG) [64]. A *grp3* mutant produces more ROS in response to flg22 or OG treatment and is enhanced resistant to *Botrytis cinerea*. The null mutant of *Autophagy 2* (*atg2-2)* is also hyper-immune and shows enhanced powdery-mildew induced cell-death that is only partially dependent on SA-signaling sectors [65]. GRP9 interacts with Cinamyl Alcohol Dehydrogenase 5 (CAD5), an essential enzyme in lignin biosynthesis and plant defenses [66,67]. LHP1-Interacting Factor 2 (LIF2) is involved in transcriptional activation of stress-responsive genes [68]. CPK28 functions as a negative immune regulator and is known to regulate BIK1 turnover via phosphorylation [69,70]. A *cpk28* mutant produces more ROS in response to a variety of PAMPs and is enhanced resistant to *PstDC3000*. Elicitor treatment results in transient dephosphorylation of an Immunoregulatory RNA-binding protein (IRR) to loosen its inhibitory association with *CPK28* transcripts causing upregulation and translation of a kinase-deficient *CPK28* splice variant thus facilitating amplified BIK1 signaling [71]. Remarkably, we detect the same intron-retained isoform of *CPK28* (Table S2) which supports the BIK1 upregulation in our ETI^HopA1pss^-like DEGs. Another common presence of IRR-regulated AS transcript is *Lesion Simulating Disease 1* (*LSD1)*, encoding a zinc finger protein implicated in cell death responses that accompany ROS elevation during immunity [72]. DAS alterations also expand to several splicing factors such as *SR30*/*34* and *CC1*. Not the least, the intron retained isoforms of *SNC1*, *SR30* and *SEN1*, identified recently in *avrRps4*-induced DAS are present in our list [44]. Overall, these data imply that ETI^HopA1pss^ feedforward amplifies PTI in an EDS1-independent manner.

As *bona fide* conserved effectors, HopA1s are anticipated to suppress PTI in susceptible plants. The identification of HopA1_pss_-interactome may reveal possible clues towards this elucidation. For one, not only several of these candidates are functionally downstream of MPK4 but also have roles in post-transcriptional or translational modulations often consorting as UPF1-interactors (such as DCP5, GRP7, PABP2/4/8, RH52, RH11, RH8 and various ribosomal proteins) [33,42]. Small heterogenous ribonucleoproteins (hnRNPs) with RNA-Recognition motifs (RRMs) such as HLP1, NTF2 and RNA-binding proteins (RBPs) GRP7 and GRP8 have activities that influence AS or closely connected events [48,49]. PABP4/8 are MPK4 substrates with likely influences on AS suppressions upon PTI [73,74]. Changes in GRP7 expression affects its own and *GRP8* AS events resulting in NMD-mediated targeting of the splice variants [75]. In context of plant defenses, GRP7/8 directs coupling of PRRs *FLS2* and *EFR* transcripts with the translational machinery [50]. When compromised in a *grp7* mutant, increased susceptibility to *PstDC3000* result [37]. The *Pseudomonas* effector HopU1 inhibits this GRP7 function on *FLS2* or *EFR* transcripts [76] and presents another classic scenario of effector suppression of PRR translations. RNA helicase RH3 occupancy on transcripts are reduced upon flg22-treatment implying that HopA1_pss_ may interfere with this PTI process [77]. DCP5 is a resident of processing (P)-bodies, cytoplasmic stores for selective mRNAs degradation and/or translational suppression [78,79]. DCP5 assists catalytic function of DCP2 in decapping mRNAs present in P-bodies. The Mediator complex subunit MED36a (also known as FIB2) was also identified in our enrichments as a HopA1_pss_-interacting protein from both plant systems. A macromolecular Mediator complex comprises of variable 20–30 subunit and coordinates interactions between bound transcription factors and the RNA polymerase II (RNA Pol II) to regulate the expression of specific downstream genes. While several MED subunits have been linked to plant defenses [80], interplay between MED19a and MED36a in immune regulations has been more recently deciphered at a molecular level [81,82]. PAMPs induce expression of a long non-coding RNA (lncRNA, termed as *ELENA*) via the PTI-signaling routes. *ELENA* inhibits MED19a-FIB2 interactions to remove FIB2 repression and facilitate MED19a-mediated transcriptional activation of *PR1*. As is therefore expected, *med19* mutant or *ELENA* knock-down plants are deficient in *PR1* induction upon PAMP exposure whereas *fib2* plants express more *PR1* and are enhanced resistant to *PstDC3000*. While the exact implications of MED36a-HopA1_pss_ interactions remains to be investigated further, nevertheless taking into account that HaRxL44 effector secreted by *Hyaloperonospora arabidopsidis*, the powdery mildew pathogen interacts with and targets degradation of MED19a suppressing SA-signaling networks to colonize plants [83], HopA1_pss_ may use a similar route via hijacking MED36a.

However, it remains to be tested whether HopA1_pst_ also interacts with these same proteins. Nevertheless, considering conserved machineries between plant and animal systems and our demonstration that both HopA1s inhibit reporter translation in cell-free extracts lead us to hypothesize that these classes of effectors likely attempt to counter loss of UPF that cause NMD-suppressions in PTI. More specially, the aimed targets likely include defense-associated transcripts including TNLs that are upregulated by PTI. Indeed, as we demonstrate that although *SNC1* is an upregulated DAS candidate in ETI^HopA1pss^-like samples, its protein levels remain unchanged (Figure 6a). In *upf1 pad4* plants, translational suppression of *RPS6* is relieved facilitating more loading into translation-proficient polysomes [41]. Contrastingly, splice variants of *RPS6* that are induced during ETI^HopA1pss^-like responses, are not loaded on polysomes indicating their translational suppression. Comprehensive RNAseq of transcripts that associate with polysomes in Col-0 versus ETI^HopA1pss^ system is necessary to reveal the diversity of cellular processes affected by HopA1 function. Alternatively, immunoblots to detect protein fate of the upregulated DAS transcripts can address the same.

**Figure 7:**
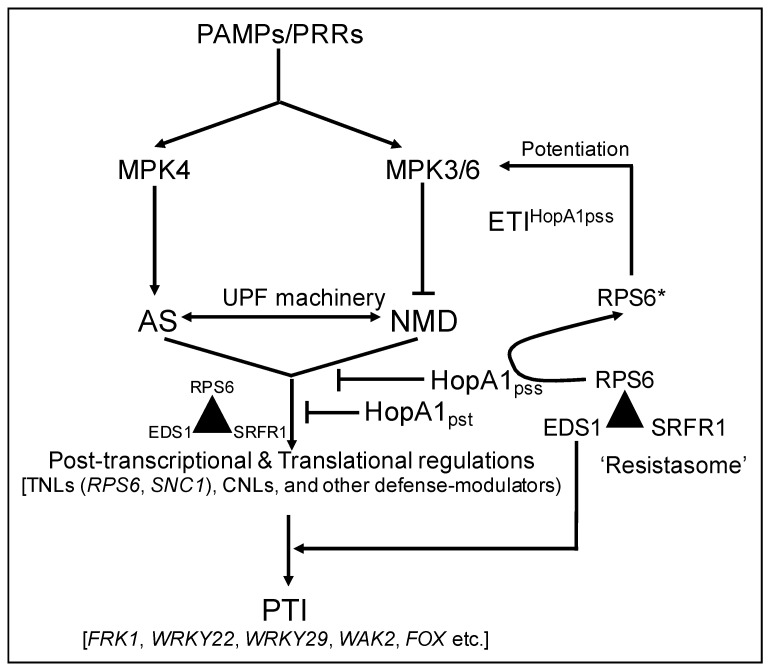
Schematic hypothetical model of HopA1 virulence, elicitation of ETI^HopA1pss^ and potentiation on PTI. PAMP perceptions by PRRs elicit PTI utilizing the MAPK (MPK3/4/6) networks to perturb AS-NMD pathway through the degradation of UPF proteins, phosphorylation of splicing and other associated factors. Upregulated splice variants of *TNL*s (RPS6, SNC1) and other defense-associated transcripts contribute to PTI signaling. Defensive overshoots are prevented by the macromolecular ‘resistasome complex (triangle) functions comprising of SRFR1, EDS1 and TNLs (RPS6). Protein-protein interactions with SRFR1 modulate uncontrolled activation of RPS6. In susceptible plants, HopA1s suppress PTI via post-transcriptional and translational inhibitions of immune-related transcripts (such as *FRK1, WRKY22, WRKY29, WAK2, FOX*) including *TNLs*. In resistant plant, resistasome sense HopA1_pss_ activities to cause activation of RPS6 (RPS6*) through unknown mechanisms. The elicited ETI^HopA1pss^ amplify PTI through MAPK3/6 signaling and other unidentified routes.

Consolidated, we present a simplified schematic model that presents virulence functions of HopA1 class of effectors and sensing of HopA1_pss_ in *RPS6* accessions (Figure 7). Under PAMP-elicitation, MPK3/6-mediated degradation of UPFs simultaneous with activated MPK4-dependent phosphorylation of AS factors relieve AS-NMD suppressions to activate expression of both positive and negative immune regulators that orchestrate PTI. The role of EDS1 in these processes remains debated although most studies and our results here place it downstream of MAPKs [84,85]. Upregulated TNL transcripts including *RPS6* presents a scenario for their activation. However, for PTI such non-specific TNL activations are unwarranted. Raxwal et al. (2020) hypothesized that RPS6 present as sentinels, is incapable of triggering defenses, unless activated by the avirulent HopA1_pss_. We surmise that this regulation is achieved at a protein-protein interaction level by the macromolecular ‘resistasome’ complex assembled by the negative immune regulator SUPPRESSOR OF rps4-RLD1 (SRFR1) [26]. In this association, PTI-upregulated RPS6 or other TNLs such as RPS4 are prevented from hyper-activation in absence of the cognate avirulent effector HopA1_pss_ or AvrRps4, respectively. A *srfr1* mutant has elevated expression of *RPS6* and other *TNL*s [26,86,87]. Countermeasures from *Pseudomonas* sp results in secretion of T3Es, and in susceptible plants HopA1 interaction with targets especially related to RNA-processes and partners of UPF1 reinforce PAMP-triggered susceptibility (PTS) by suppressing the expression of defense-associated genes through post-transcriptional and translational regulations. The intricacies of these processes await further studies. In resistant accessions, HopA1_pss_ is sensed by the resistasome and lead to activation of RPS6 through unknown mechanisms [26]. Activated RPS6 elicits ETI through potentiation on PTI signaling networks. We have identified that immuno-enrichments of SRFR1 co-elutes several HopA1_pss_-interacting proteins implying the physical presence of the resistasome at the same cellular locale as HopA1_pss_ (data not shown). We continue to pursue further investigations on these aspects to provide molecular link of resistasome functions in HopA1_pss_ surveillance and regulation of immunity. In summary, our findings here present promising avenues to explore host manipulations by HopA1 effectors, evasion of host detection and its interception modes in resistant plants.

## 4. Material and Methods

### 4.1. Plasmids Constructs and Generation of Transgenic Plants

The construction of *p*DONR201-HopA1_pss_ have been described earlier [26]. Similarly, HopA1_pst_ sequence was cloned into *p*DONR201 via gateway methodology. Both HopA1_pss_ and HopA1_pst_ were subsequently cloned into Myc-pBA vector to generate Myc-HopA1_pss_ and Myc-HopA1_pst_, respectively. To generate Dex-inducible HopA1_pss_ or HopA1_pst_ clone, the *p*TA7002 vector [88] was used. First, a cMyc-epitope tag was introduced at the *Xho*I-*Spe*I site using overlapping oligos (Stg *Xh*oI-*Xba*I-Myc/ComStg*Xho*I-*Xba*I-Myc) that recreated the *Xho*I site, destroyed the native *Spe*I site in the original vector and created an internal *Spe*I site 3′-to the single cMyc epitope sequences. This vector was termed *p*TA7002-Myc. HopA1_pss_ or HopA1_pst_ sequences were amplified as *Xho*I-*Spe*I fragment and cloned into similar restriction sites of *p*TA7002-Myc. The above generated binary vectors were electroporated into *Agrobacterium tumefaciens* strain GV3101 and used in the indicated assays. To generate His-tagged expression clones of HopA1_pss_, HopA1_pst_ or GFP, the respective *p*DONR201 clones were used in a gateway reaction with *p*DEST17 vector (Thermo Fisher, Boston, MA, USA). Confirmed clones were transformed into *E. coli* BL21 AI (Arabinose inducible; Thermo Fisher, Boston, MA, USA) cells for recombinant protein expression and purification. Sequence of all primers used here are listed in Table S7.

Floral-dip transformation of *A. thaliana* with *Agrobacterium* strains containing *p*TA7002-HopA1_pss_ or *p*TA7002-HopA1_pst_ binary was performed according to the earlier report [89]. Transgenic plants were selected on hygromycin (20 μg/mL) containing 0.5x MS-agar plates and propagated for further generation for homozygosity of the transgene.

### 4.2. Plant Growth Conditions

*N. benthamiana* and *N. tabacum* cv xanthi plants were grown in a controlled-environment chamber (24 °C with 70% RH) under 16 h of light (100 μmol μm^−2^s^−1^). *A. thaliana* plants were also maintained under same conditions for propagation and seed bulking. For HR assays, the plants were grown under short-day conditions (8 h light:16 h dark) with other parameters similar as earlier.

### 4.3. Transient Agrobacterium-Mediated Expression Assays in N. benthamiana and N. tabacum Plants

Transient expression assays in *N. benthamiana* or *N. tabacum* plants were performed as described previously [26]. Briefly, *Agrobacterium* strains containing the indicated binary vector was cultured in liquid Luria-Bertani (LB) medium supplemented with appropriate antibiotics. Bacterial cells were harvested by centrifugation for 10 min and resuspended in induction buffer [10 mM MgCl_2_, 10 mM MES (pH 5.6) with 100 µM acetosyringone (Sigma-Aldrich, St. Louis, MO, USA)] and incubated for 5–6 h at room temperature. Bacterial suspensions were then adjusted to OD_600nm_ = 0.6 (~6 × 10^8^ CFU/mL) and infiltrated into fully expanded leaves of *N. benthamiana* or *N. tabacum* leaves using a needleless syringe. The inoculated plants were moved to the growth chamber for the next 24–48 h, as indicated, before analysis.

### 4.4. Dexamethasone Treatments

Dexamethasone (Dex) (Sigma-Aldrich, St. Louis, MO, USA) 30 mM stock was made in DMSO. For plant applications, 30 μM working solution was made in induction buffer containing 0.01% Tween-20 and sprayed on the HopA1_pss_-expressing transgenic or on control plants. Samples were harvested at indicated time-points post-induction (hpin) for analysis.

### 4.5. Protein Extraction and Immunoblot Analysis

Protein detection from transient expression assays in *N. benthamiana* were performed at 48 hpi. Protein extractions from transgenic Arabidopsis plants were performed at 12- or 24 hpin, as indicated. Briefly, three leaf discs (19 cm diameter each) were excised from the infiltrated area and homogenized in 6 M Urea solution. After centrifugation to remove cell debris, 1x Laemmli buffer was added to the supernatant. Protein extractions for MPK3/6 or SNC1 was performed as reported earlier [90,91], respectively. Proteins were resolved on SDS-PAGE gel and blotted onto PVDF membrane. The membrane was blocked with 5% non-fat skim milk and immunoblots were performed with indicated primary [anti-Myc (Biobharati, Kolkata, WB, India), anti-Actin C3 (Abiocode, Agoura Hills, CA, USA), anti-PR1 (Agrisera, Vännäs, Sweden), anti-EDS1 (Agrisera), anti-SNC1 (Abiocode) or anti-MPK3/6 active pTEpY (Promega, Madison, WI, USA)] and appropriate HRP-conjugated secondary antibodies. The blots were developed using ECL™ Prime western blotting system (GE Healthcare, Chicago, IL, USA) and visualized in ImageQuant™ LAS 4000 biomolecular imager (GE Healthcare).

### 4.6. Electrolyte Conductance Assay

Electrolyte leakage assays were performed according to Johansson et al. [92]. In brief, ten leaf disks from the inoculated area in at least three different leaves were excised with a 0.6 cm-diameter cork-borer. Samples were placed in a 15 mL 10 mL of distilled water. The tubes were shaken at 200 rpm at room temperature for 1 h. After removing all leaf discs, the conductivity of the bathing solution was measured using TDS meter with ATC (range 10/1990 ppm).

### 4.7. RNA Extraction and Gene Expression Analysis by qRT-PCR

Total RNA was isolated after transient expression or Dexamethasone treatment from indicated plants with RNAiso Plus (Takara-Bio, Otsu, Shiga, Japan). RNA was reverse transcribed (iScript™ cDNA Synthesis Kit; Bio-Rad, Hercules, CA, USA) according to manufacturer’s instructions. All qPCR primers used in this study are listed in Table S5. qPCRs were performed in Quant Studio 6 Flex Real-Time PCR system (Applied Biosystems, Waltham, MA, USA) with 5X HOT FIREPol^®^ EvaGreen^®^ qPCR Mix Plus (ROX) (Solis BioDyne, Tartu, Estonia) according to the manufacturer instructions. All qPCR experiments were repeated at least twice with three replicates (*n* = 3). Relative expression was calculated according to the PCR efficiency^^−ΔΔCt^ formula. Expression differences were normalised to the invariant internal standard *MON1* (for Arabidopsis) [93], *Actin* (for *N. benthamiana*) or *EF1* (for *N. tabacum*) and reported as fold-change relative to mock, DEX^−^ treatment or Col-0 as indicated.

### 4.8. Immunoprecipitation

Total protein extracts were prepared from infiltrated *N. benthamiana* leaves (at 48 hpi) or transgenic Arabidopsis plants (12 hpin) by homogenizing in liquid nitrogen and resuspension in lysis buffer [10% glycerol, 25 mM Tris-HCl (pH 7.5), 150 mM NaCl, 2% PVPP, 10 mM DTT, 1X protease inhibitor, 1 mM PMSF]. Cell debris was removed by centrifugation and supernatant precleared for 1 h with IgG-conjugated (as non-specific binding control) beads and then incubated for 3 h with anti-Myc, or anti-HA antibody-conjugated beads (Sigma-Aldrich) at 4 °C under gentle rotation. After extensive washing with lysis buffer, the bound proteins were eluted by boiling in 1x Laemmli buffer. Eluted proteins were separated by SDS–PAGE and immunoblotted with indicated primary and secondary antibodies. The blots were developed as earlier.

### 4.9. Sample Preparation and In-Gel Trypsin Digestion

The sample preparation protocol for mass spectrometry analysis described earlier [93,94] was followed with minor modifications. After confirmation of immunoprecipitation enrichments, the respective eluates were electrophoresed on 8% SDS-PAGE for 15–20 min for the total proteins to enter the resolving gel. The gel was stained with Coomassie brilliant blue (CBB R-250) and then de-stained with de-staining solution [50% water, 45% methanol and 5% Glacial acetic acid] for 3–4 h. Protein bands in the gel were cut into 1 mm^3^ pieces using sterile surgical blades and collected in fresh 1.5 mL micro-centrifuge tubes. Gel pieces were washed with washing solution [50:50 solution of Acetonitrile (ACN): Water in 50 mM Ammonium bicarbonate (ABC)] thrice to remove CBB stain completely. The gel pieces were then dehydrated by adding 500 μL of 100% ACN solution for 10 min and dried using speed-vac centrifuge at room temperature (RT). Disulfide bonds were reduced by addition of alkylation solution [10mM DTT in 50 mM ABC] and incubated at 60 °C for 30 min. Then, 100 μL of reduction solution [55 mM IAA in 50 mM ABC] was added and were incubated for 30 min at RT in dark. Alkylation solution was removed and gel pieces were washed with 500 μL washing solution. Gel pieces were dehydrated again with 500 μL of 100% ACN for 10 min. Residual ACN was removed and gel pieces were dried completely using a speed-vac centrifuge. Samples were incubated with Trypsin (Promega) at final protease: protein ratio of 1:20 (*w/w*) for in-gel digestion at 37 °C for 16 h. Digested peptides were extracted using gradient of 20% to 80% ACN diluted in 0.1% formic acid (FA). Extraction solution having gel pieces were sonicated for 5 min in ultra-sonicator water bath to achieve maximum recovery of peptides. The pooled extracts were dried using speed-vac centrifuge. The peptides were desalted with C18 Tips (Thermo Scientific), 100 μL bed according to manufacturer instructions and peptides were eluted in 100 μL of 75% ACN in 0.1% FA. Eluates were dried using speed-vac centrifuge. Vacuum dried peptides were resuspended in 10 μL 2% ACN in 0.1% FA and subjected to MS/MS using Triple TOF^®^ 5600+ (ABSciex, Framingham, MA, USA) mass spectrometer instrument.

### 4.10. Data Processing and Analysis

For HopA1-proteome analysis *in planta*, raw MS data files were searched and peptide sequences were assigned using the MASCOT software (version 2.7.0, Matrix Science) [95] and Protein Pilot Software against the *Arabidopsis thaliana* protein database www.NCBI.nlm.NIH.gov/RefSeq/ (accessed on 20 May 2021) and *Nicotiana* database https://solgenomics.net/organism/Nicotiana_benthamiana/genome (accessed on 20 May 2021). As the *N. benthamiana* protein database is not completely annotated, the MS raw data files were also searched against the *Arabidopsis* database to identify corresponding orthologous protein targets. HopA1 bait protein sequence was externally searched in raw MS data file. For further analysis, proteins with minimum of one unique peptide with ≥95% confidence level were considered for analysis. For PPI network construction and GO analysis, the STRING version 11.0 https://string-db.org (accessed on 21 May 2021) was used.

### 4.11. Protein Purifications

The recombinant plasmids (*p*DEST17-GFP, *p*DEST17-HopA1_pss_, pDEST17-HopA1_pst_) were separately transformed into the expression host *E. coli* BL21 AI cells. Expression and purification of His_6_-fused proteins was carried out according to the manufacturer’s protocol (Qiagen, Venlo, Netherlands). A single colony was grown in LB medium containing appropriate antibiotics to an OD_600_ of 0.6, following which isopropyl-β-d-1-thiogalactopyrannoside (IPTG, 0.5 mM final) and L-arabinose (0.2% final) was added into the medium and incubated with shaking at 25 °C overnight. The bacterial cells were pelleted and resuspended in lysis buffer [100 mM Tris-HCl, 300 mM NaCl, 50 mM NaH_2_PO_4_, 10 mM imidazole, pH 8.0] and sonicated. Soluble supernatant protein was purified using Ni^2+^-NTA resin (Qiagen) under native conditions as described in the manual. Elution of the bound recombinant His_6_-tagged proteins was performed with lysis buffer containing 250 mM imidazole. Imidazole was removed by overnight dialysis using Spectra/Por3 Dialysis membrane (VWR). The extent of purity of the recombinant protein was determined on SDS-PAGE using Anti-His antibodies (Biobharati).

### 4.12. In Vitro Transcription and Translation-Coupled Luciferase Expression Assays

Cell-free assays for *Luciferase* (*LUC*) expression was performed using rabbit reticulocyte lysate system according to manufacturer’s instructions (Promega). Purified His-GFP (negative control), His-HopA1_pss_ or His-HopA1_pst_ at equimolar concentrations (1 μM) were added to the reaction mix. After incubation of reaction at 30 °C for 90 min, the luminescence of *LUC* expression was checked using luciferase assay system (Promega) according to instructions provided and measured using a luminometer (Promega). Similar experiments with heat-denatured HopA1s were performed with 0.5 μM proteins. Expression was reported as relative luminescence units (RLU) relative to reaction in absence of the *LUC* plasmid. Transcription efficiencies of *LUC* was determined by semi-quantitative RT-PCR with endogenous *β-globin* gene expression as the reference.

### 4.13. RNA Sequencing and Bioinformatics Analysis

Total RNA was isolated from HopA1_pss_-expressing transgenic line post DEX treatment (at 12 hpin). After RNA quality confirmation, two biological replicates were sequenced by next generation sequencing. The following parameters were performed from the fastq file, base quality score distribution, sequence quality score distribution, average base content per read, GC distribution in the reads, PCR amplification issues, over-represented sequences and adapter trimming. Based on quality report of fastq files we trimmed the sequence appropriately to only retain high quality sequence for further analysis. In addition, the low-quality sequence reads were also excluded from the analysis. The adapter trimming was performed using Trimmomatic (v-0.36) http://www.usadellab.org/cms/?page=trimmomatic (accessed on 3 June 2021). For the RNAseq analysis non-polyA-tailed RNAs mitochondrial and ribosomal-encoded RNAs, transfer RNAs, adapter sequences were removed. Contamination removal was performed using Bowtie2 (2.2.4). TAIR 10 (*FTP://FTP.ARABIDOPSIS.ORG/HOME/TAIR/SEQUENCES/WHOLE_CHROMOSOMES/*) version of the genome was used for analysis. The paired-end reads are aligned to the reference *Arabidopsis thaliana* genome (TAIR10). Alignment was performed using HISAT2 (2.1.0) [95,96]. The aligned reads were used for estimating expression of the genes. The raw read counts were estimated using feature Count (1.5.2). Read count data were normalized using DESeq2 [97]. The ratio of normalized read counts for treated over control was considered as the fold-change. Transcripts were first filtered based on the *p* value (≤ 0.05). A distribution of these log_2_ (fold-change) values were found to be normally distributed. Those genes which were found to have −1 ≥ log_2_(fold-change) ≥ 1 were considered statistically significant and differentially expressed (DEGs). Gene ontology (GO) and pathway annotations for DEGs were carried out using AraCyc software from the TAIR database. Alternate-splicing (DAS) analysis was performed using rMATS software [43] using paired replicates (DEX^+^ R1 and R2; DEX^−^ R1 and R2) of each sample. The likelihood-ratio test is adopted to calculate statistical significance value between paired replicates. Sashimi plots were generated for all the significant DAS events identified in the presence of HopA1_pss_ effector.

### 4.14. GO Term Analysis of RNAseq and Proteomics Data

For GO enrichment analysis of RNAseq and HopA1_pss_-protein interactome data ShinyGO server was used [31]. The analysis was functioned with the false discovery rate of <0.01. Further, Revigo plot [98] was generated using GO term for biological process enrichment with allowed 0.9 similarity to each GO term.

### 4.15. Polysome Assay and qRT PCR

Polysome extraction assay was performed according to protocol as described [93]. Briefly, ~100 mg tissues from three-weeks-old *DEX: HopA1_pss_-Myc eds1-2* with and without DEX (30 µM) treatment were ground to a fine powder in liquid nitrogen, resuspended in polysome extraction buffer (PEB) [200 mM Tris-HCl, pH 9.0, 25 mM EGTA, 200 mM KCl, 36 mM MgCl_2_, 5 mM dithiothreitol (DTT), 50 mg/mL cycloheximide, 50 mg/mL chloramphenicol, 0.5 mg/mL heparin, 1% (*v/v*) Triton X-100, 1% (*v/v*) Tween 20, 1% (*w/v*) Brij-35, 1% (*v/v*) Igepal CA-630 or NP-40, 2% (*v/v*) polyoxyethylene and 1% (*w/v*) deoxycholic acid] and clarified by centrifugation at 16,000× *g* for 20 min at 4 °C. Supernatant were overlaid on 1.6 M sucrose cushion and ultra-centrifuged at 170,000× *g* for 18 h at 4 °C. The polysome pellet was resuspended in DEPC (diethylpyrocarbonate)-treated water. RNAs from the pellet fractions were isolated with RNAiso plus, reverse transcribed and qPCR performed as described earlier.

### 4.16. Statistical Analysis

Pairwise Student’s *t*-test was performed to check significance and denoted by one, two and three asterisks indicating *p*-value < 0.05, <0.01 and 0.001, respectively, for gene-expression related experiment. GraphPad PRISM (version 7.0a) https://www.graphpad.com/scientific-software/prism/ (accessed on 16 May 21) software was used to perform statistical analysis and for making the graphs. Adobe Illustrator CC version 2019, https://www.adobe.com/in/products/illustrator.html (accessed on 12 June 21) software was used to make final images.

## Data Availability

Raw sequencing data generated in this study is available at Sequence Read Archive (NCBI) under the accession numbers: SRR14139156, SRR14139155, SRR14139154, SRR14139153.

## Supplementary Materials

The following are available online at https://www.mdpi.com/article/10.3390/ijms22147440/s1, Figure S1: In *N. benthamiana*, transient expression of HopA1_pss_ elicits stronger defenses than HopA1_pst_, Figure S2: HopA1_pss_ over-expression elicits *EDS1*-independent *PR1* upregulation, Figure S3: MPK3/6 phosphorylations are enhanced by HopA1_pss_ expression *in planta,* Figure S4: Differentially expressed genes (DEGs) in ETI^HopA1pss^-like responses, Figure S5: GO enrichment categorizations of DEGs in Dex^+^ HopA1_pss_-expressing transgenic plants in terms of biological processes, Figure S6: ETIHopA1pss-like elicitation upregulates expression of PTI and other defense-associated transcripts, Figure S7: REVIGO plots of biological process GO enrichment clusters present in DEGs and DAS of Dex^+^ HopA1_pss_-expressing transgenic plants, Figure S8: Classification of DAS events in ETI^HopA1pss^-like responses, Figure S9: Alternative splicing of transcripts are perturbed during ETI^HopA1pss^-like responses, Figure S10: Schematic outline of HopA1_pss_ immuno-enrichment steps from plants, Figure S11: STRING protein-protein interaction network of co-eluting proteins from HopA1_pss_ immuno-enrichments, Figure S12: Translational suppression activities are abolished for heat-denatured HopA1s, Table S1: Upregulated *R* genes and their categorizations identified in ETI^HopA1pss^, Table S2: Unique differentially alternatively spliced (DAS) candidates and their event classifications identified in ETI^HopA1pss^, Table S3: List of *A. thaliana* proteins enriched with HopA1_pss_ (provided as a separate Excel file), Table S4: List of *N. benthamiana* proteins enriched with HopA1_pss_ (provided as a separate Excel file), Table S5: Common HopA1_pss_-interacting/co-eluted proteins identified by mass spectrometry in *N. benthamiana* and *A. thaliana.* Table S6: Structural comparison of HopA1s with PDB entries, Table S7: List of oligonucleotide primers used in this study.
