# Supplementary material for: HopA1 Effector from Pseudomonas syringae pv syringae Strain 61 Affects NMD Processes and Elicits Effector-Triggered Immunity
Source: Int J Mol Sci. 2021 Jul 12;22(14):7440. doi: 10.3390/ijms22147440 (PMC8306789; doi:10.3390/ijms22147440)
Supplement: Supplementary file 1 [file ijms-22-07440-s001.zip › Supplementary Information.pdf]

Supplementary Information:

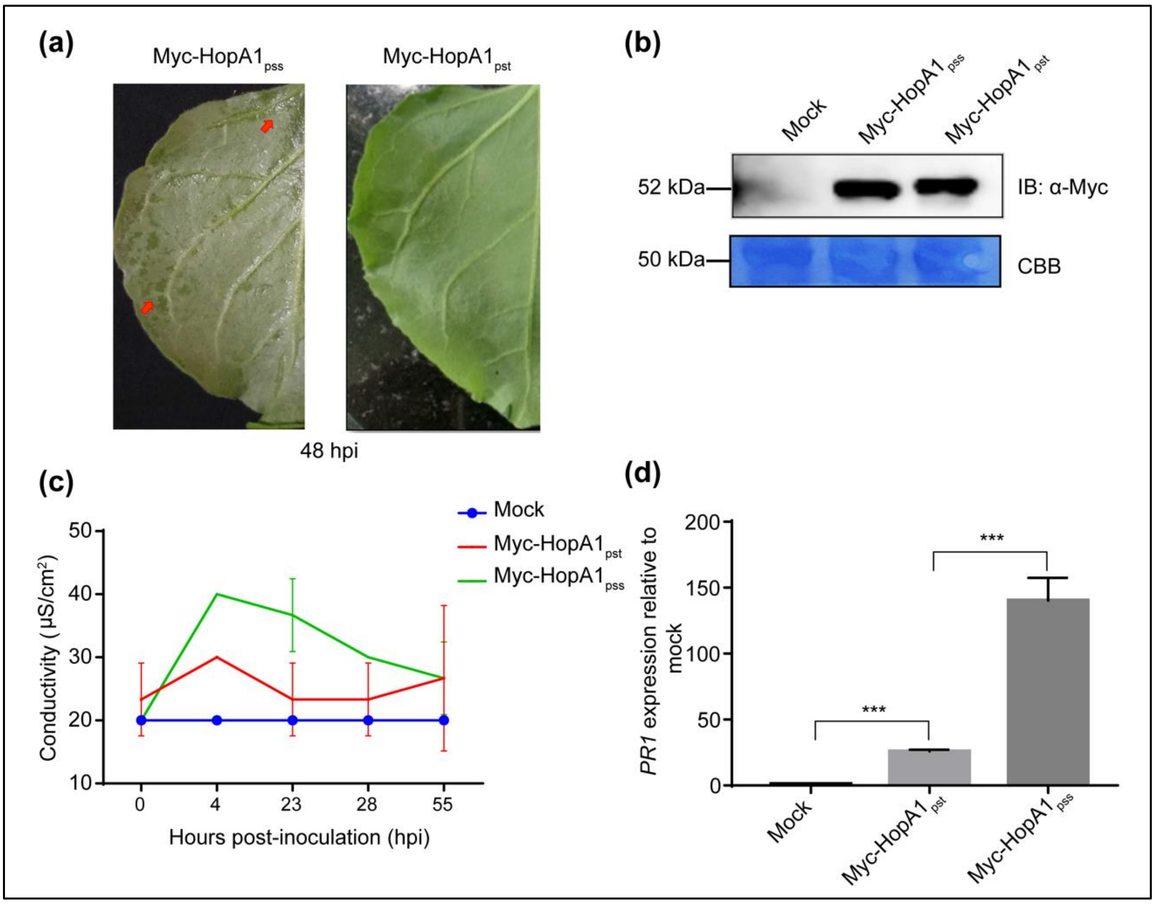

Supplementary Figure S1: In *N. benthamiana*, transient expression of HopA1$_{pss}$ elicits stronger defenses than HopA1$_{pst}$. Buffer alone (mock) or agrobacterium strains expressing Myc-HopA1$_{pss}$ or Myc-HopA1$_{pst}$ were infiltrated into fully expanded leaves. (**a**) Infiltrated leaf imaged at 48-hours post-infiltration (hpi). (**b**) Expression levels of Myc-HopA1$_{pss}$ or Myc-HopA1$_{pst}$ proteins determined at 48hpi via immunoblotting with anti-Myc antibodies. (**c**) conductance assay on infiltrated leaf dics at indicated time points (hpi). (**d**) Expression levels of *PR1* transcripts at 48hpi. Ponceau S stained membrane shows comparable protein loading between samples. Values are mean $\pm$ SD (n=10 for conductance assays, n=3 for qRT-PCRs). Statistical analysis shown is according to Student's *t*-test ($p^{**}<0.01$).

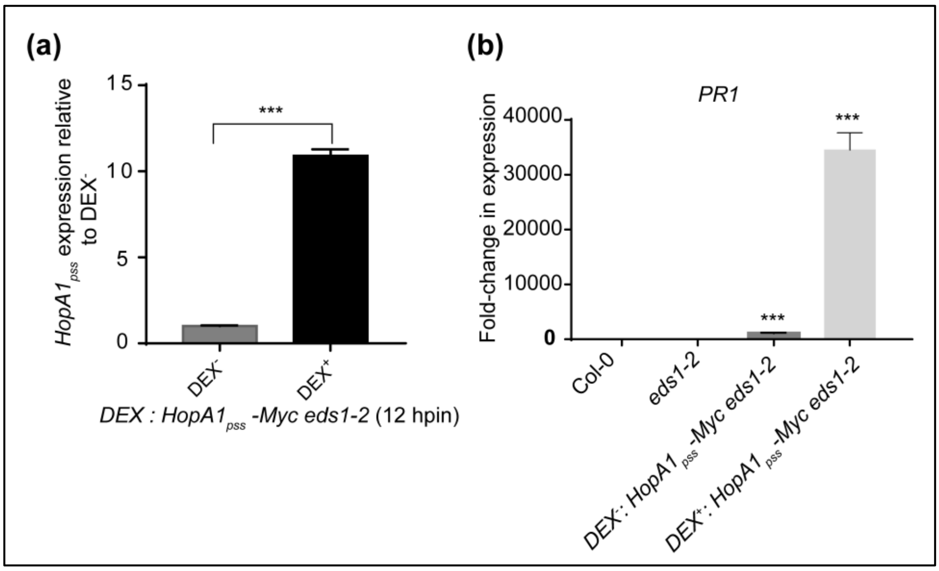

Supplementary Figure S2: HopA1$_{pss}$ over-expression elicits *EDS1*-independent *PR1* upregulation. *Dex:HopA1$_{pss}$-Myc eds1-2* transgenic plants without (Dex$^-$) and with (Dex$^+$) dexamethasone (Dex)-treatment at 24-hours post-induction (hpin). (**a**) *HopA1$_{pss}$*, and (**b**) *PR1* transcript levels at 12- or 24-hpin. Data is representative of mean $\pm$ SD (n=3). Pairwise Student's *t*-test was used to determine statistical significance (*p****<0.001).

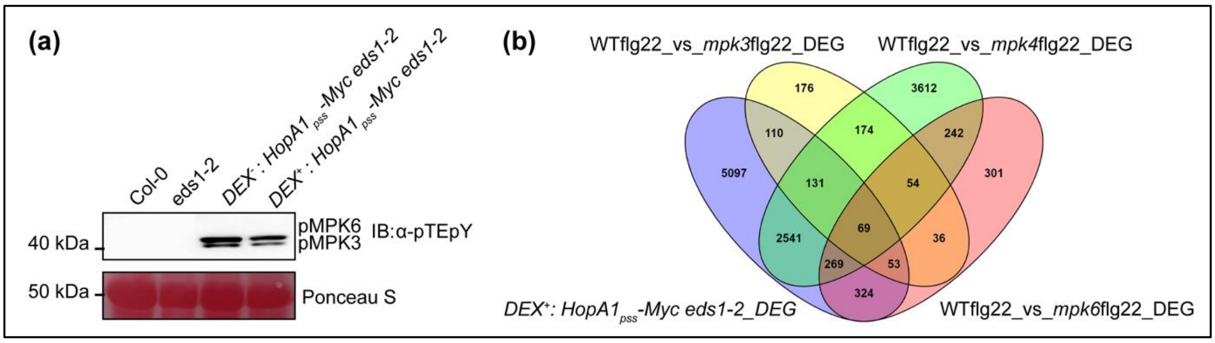

Supplementary Figure S3: MPK3/6 phosphorylations are enhanced by HopA1$_{pss}$ expression *in planta*. (**a**) Immunoblot of total protein extracts from Col-0, *eds1-2*, Dex$^-$ and Dex$^+$ HopA1$_{pss}$-expressing transgenic plants at 12hpin with anti-active MAPK pTEpY polyclonal antibodies. Positions of phosphorylated MPK3 and MPK6 are indicated. Ponceau S stained membrane represents comparable protein loading across samples. (**b**) Venn diagram of DEG overlaps of Dex$^+$ HopA1$_{pss}$ plants with similar datasets from flg22-elicited *mpk3*, *mpk4*, or *mpk6* mutants versus flg22-WT (from Bazin *et al*., 2020).

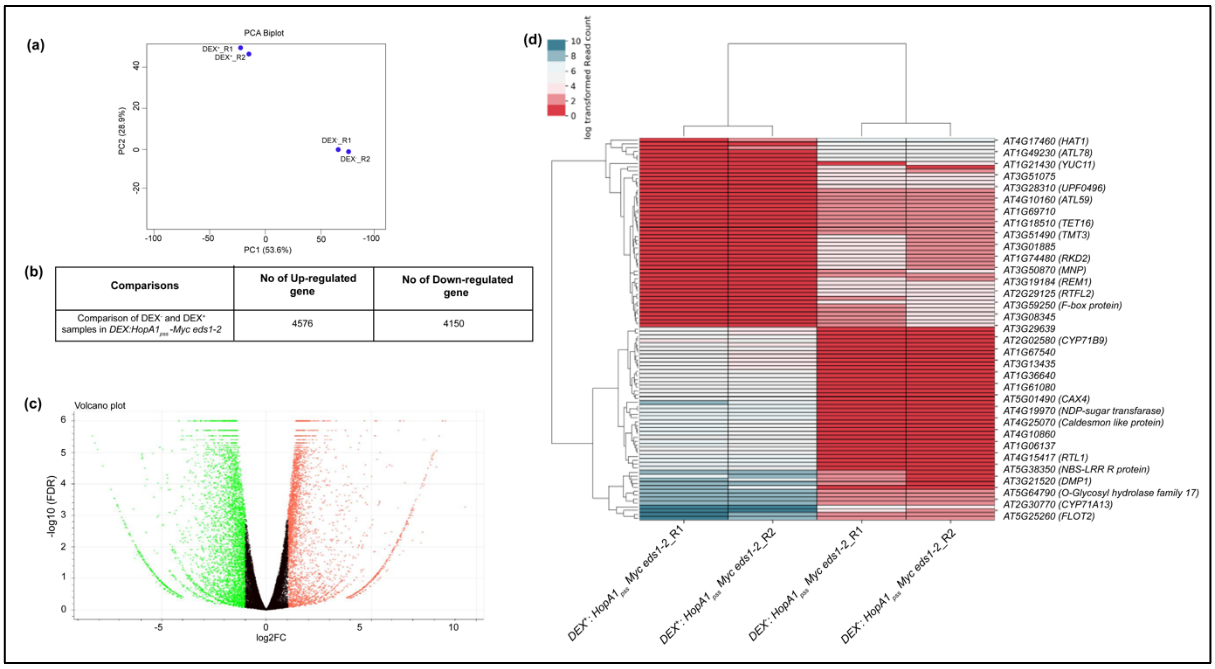

Supplementary Figure S4: Differentially expressed genes (DEGs) in ETI^{HopA1pss}-like responses. (**a**) Scatter plot of Principal Components (PCs) of the data from all samples. Each point represents an RNAseq sample. Samples with similar gene expressions are closer in the two-dimensional space. Replicate 1 and 2 (Dex^{+}_R1 and _R2) of Dex-treatment or untreated (Dex^{-} _R1 and _R2) are marked. (**b**) Number of DEGs in the RNAseq data. (**c**) Volcano plot of DEGs. (**d**) Heatmaps of top 50 upregulated and top 10 downregulated candidate genes in replicate samples processed for RNAseq.

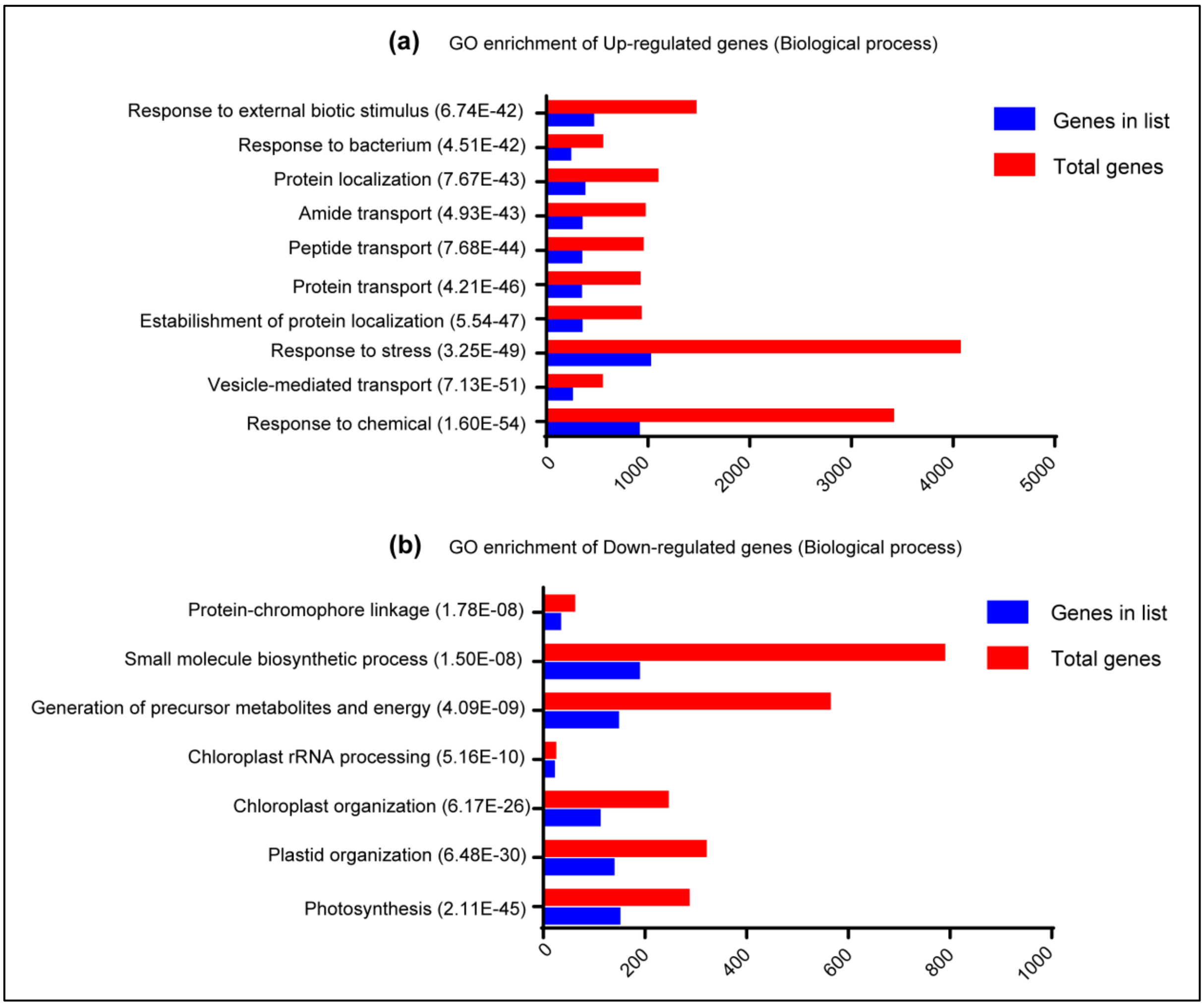

Supplementary Figure S5: GO enrichment categorizations of DEGs in Dex^{+} HopA1$_{pss}$-expressing transgenic plants in terms of biological processes. (**a**) Upregulated, and (**b**) downregulated DEGs.

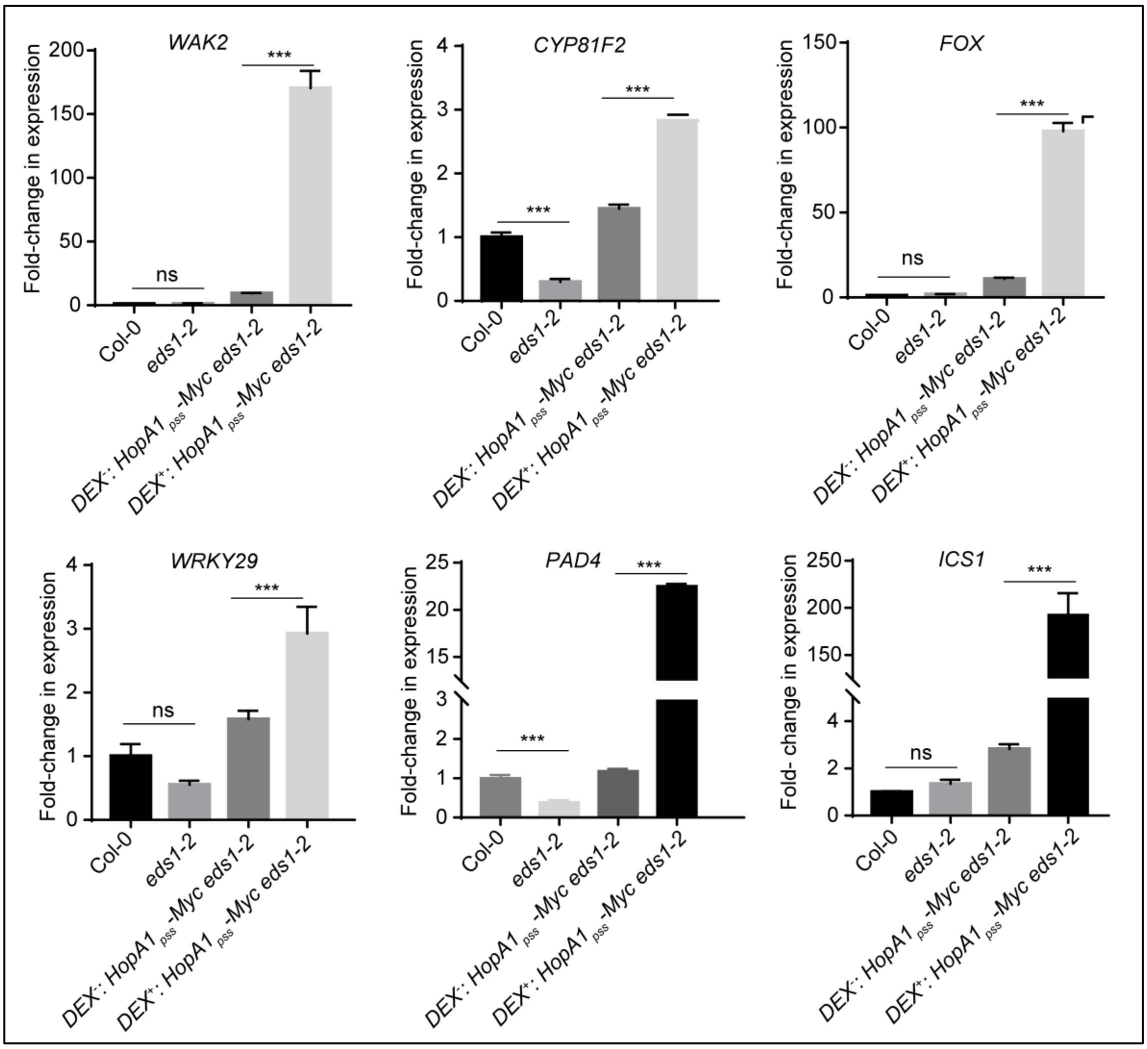

Supplementary Figure S6: ETI^{HopA1pss}-like elicitation upregulates expression of PTI and other defense-associated transcripts. Expression levels of *WAK2, CYP81F2, FOX, WRKY29*, *PAD4* and *ICS1* in HopA1$_{pss}$-expressing transgenic at 12hpin or uninduced control, Col-0 and *eds1-2* plants. Data is mean $\pm$ SD (n=3) and represented as fold-change relative to Col-0. Statistical significance is with Student's *t*-test and show pairwise comparisons to Col-0 levels (*p****<0.001).

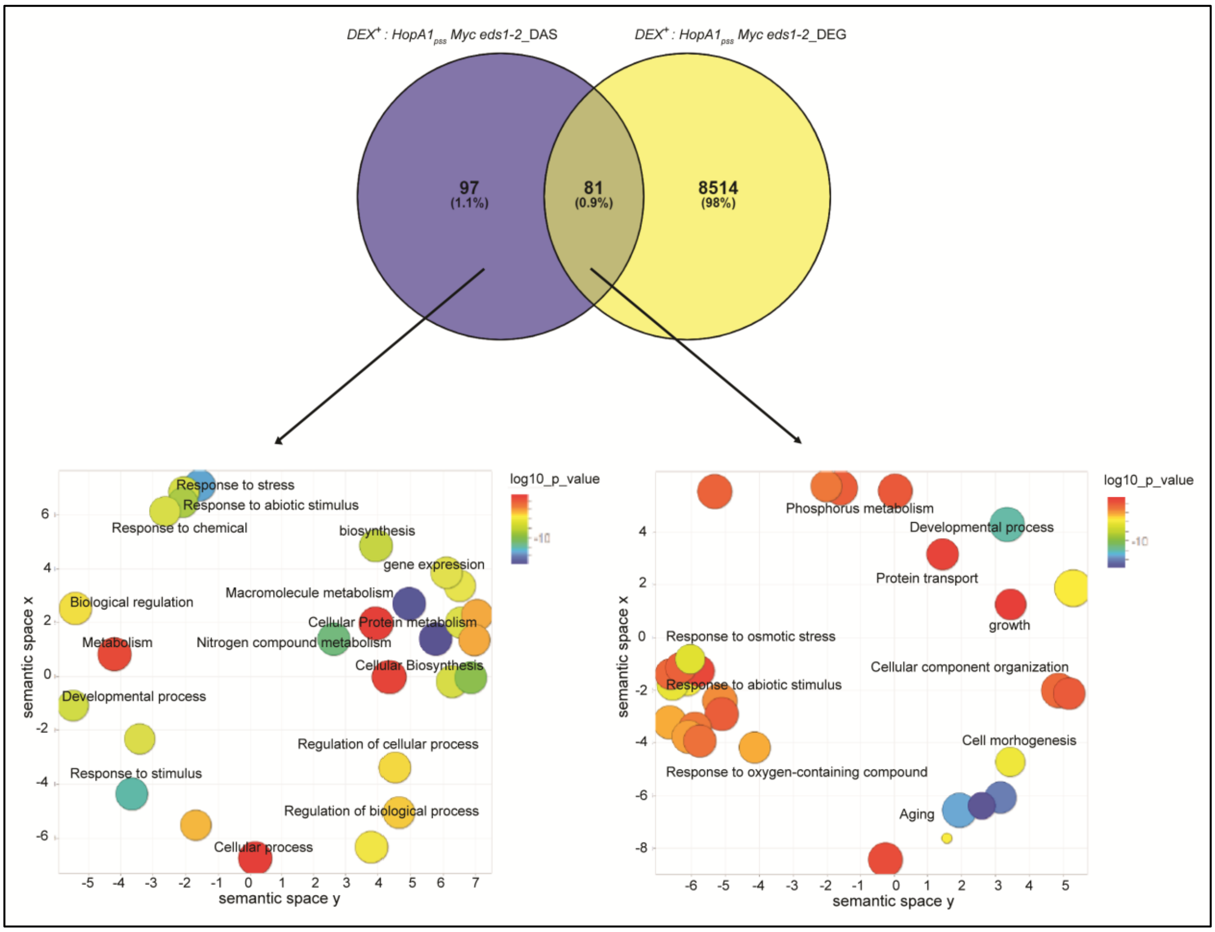

Supplementary Figure S7: REVIGO plots of biological process GO enrichment clusters present in DEGs and DAS of Dex+ HopA1$_{pss}$-expressing transgenic plants. Labeling is shown only for highest significant categorizations.

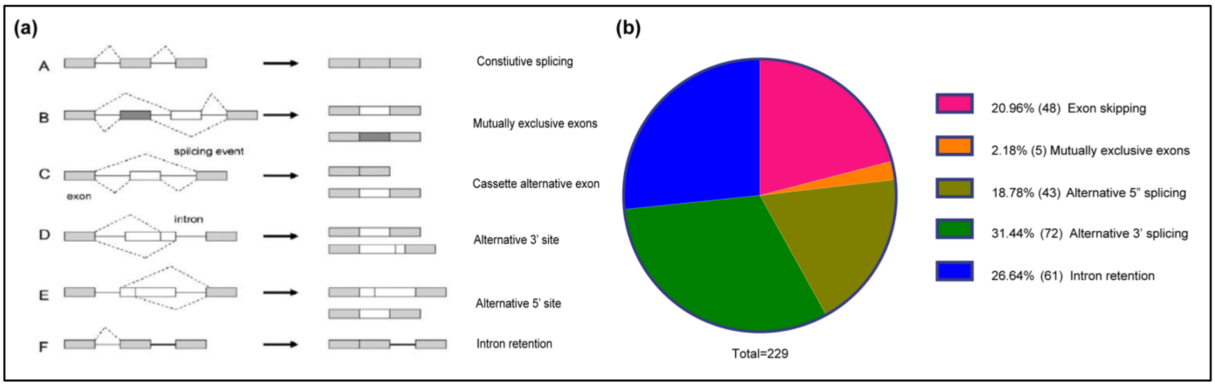

Supplementary Figure S8: Classification of DAS events in ETI^{HopA1pss}-like responses. (**a**) Schematic representation of AS events. (**b**) Pie chart showing enrichment of DAS according to AS event categorizations.

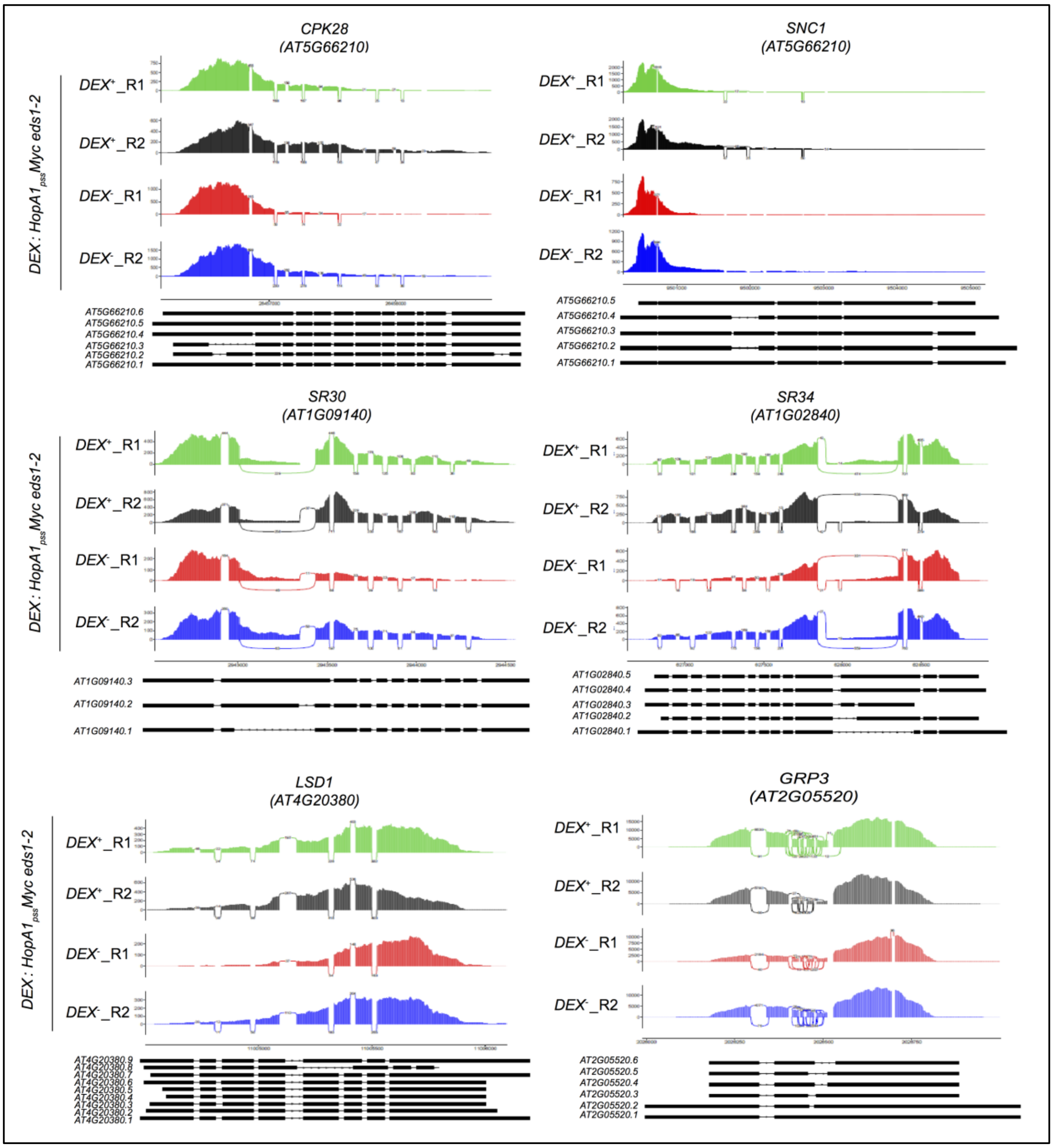

Supplementary Figure S9: Alternative splicing of transcripts are perturbed during ETI^{HopA1pss}-like responses. Sashimi plot showing representation of DAS events in selective immunity-associated genes *CPK28*, *SNC1*, *SR30*, *SR34*, *LSD1* and *GRP3* identified in ETI^{HopA1pss}- like DAS datasets.

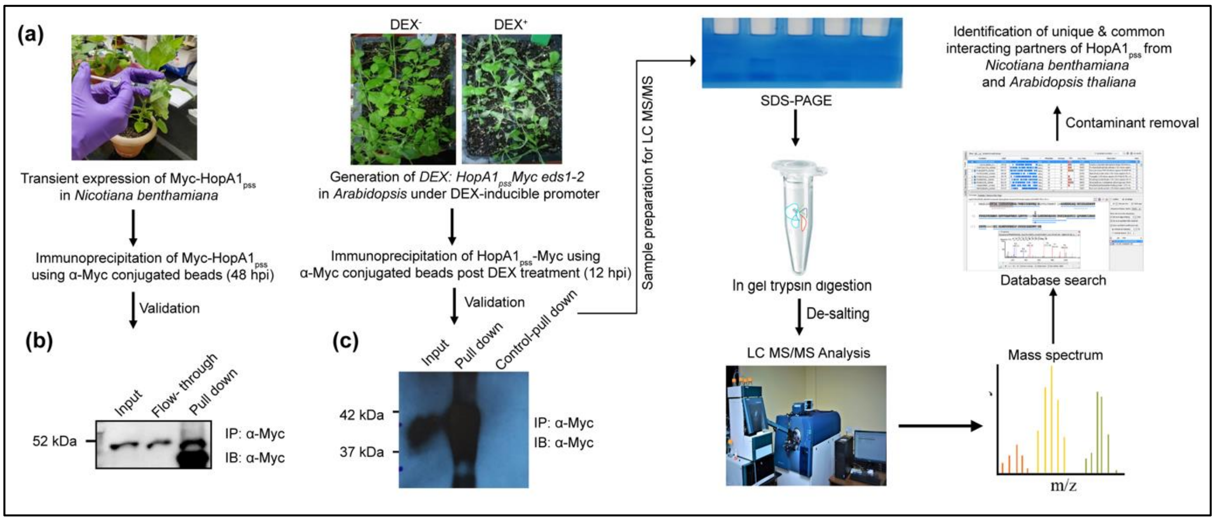

Supplementary Figure S10: Schematic outline of HopA1$_{pss}$ immuno-enrichment steps from plants. (**a**) Sample harvesting and processing from transgenic Arabidopsis (at 12hpin) or transiently expressing *N. benthamiana* plants (at 48hpi). Anti-Myc immunoblots of enriched samples from (**b**) *N. benthamiana*, and (**c**) HopA1$_{pss}$-expressing Arabidopsis submitted for LC-MS/MS. Migration position of molecular weight standards (in kDa) are indicated.

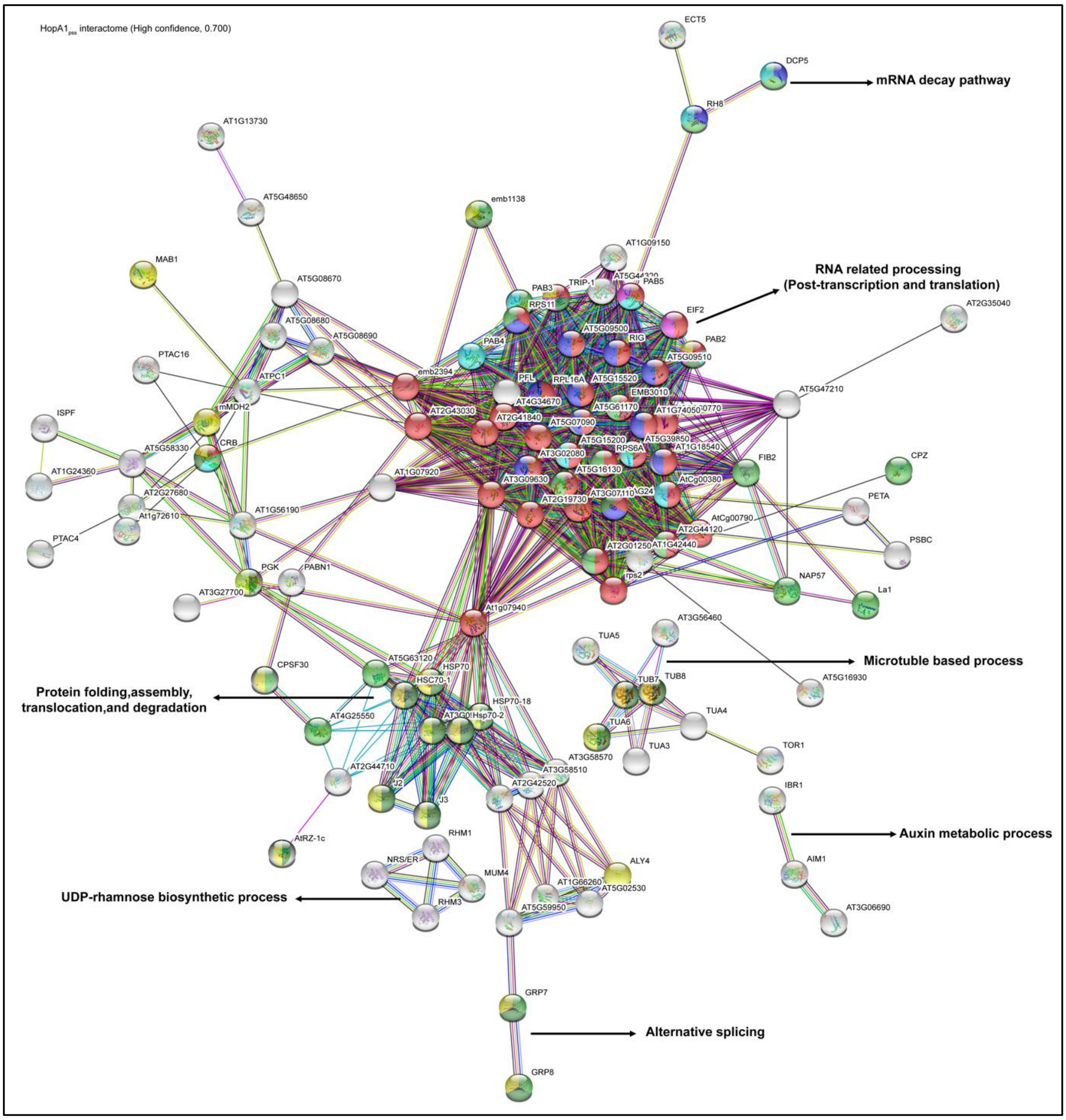

Supplementary Figure S11: STRING protein-protein interaction network of co-eluting proteins from HopA1$_{pss}$ immuno-enrichments. Functional involvement to cellular processes are indicated next to the protein clusters.

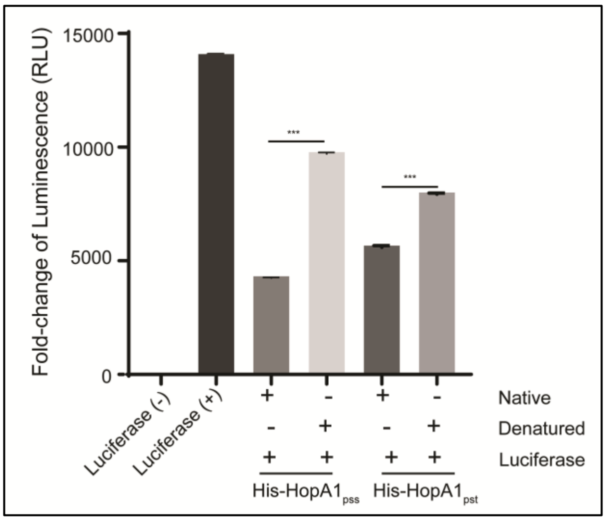

Supplementary Figure S12: Translational suppression activities are abolished for heat-denatured HopA1s. Equimolar amounts of $HopA1_{pss}$ or $HopA1_{pst}$ were either heat-denatured or not and used in *in vitro* transcription-translation assays determining the expression of *LUC* reporter. Expression is measured as RLU (relative luminescence units) and represented as fold-change relative to control (Luc-) reaction. Values are mean $\pm$ SD of three biological replicates (n=3). Statistical analysis is with student's *t*-test (*p* ***< 0.001).

Supplementary Table S1: Upregulated *R* genes and their categorizations identified in ETI^{HopA1pss}

**TIR-NBS-LRR**	**CC-NBS-LRR**	**TIR-NBS**	**TIR-Domain family**	**NB-ARC**	**LRR and NB-ARC domain**	**NBS-LRR**	**Putative**
AT1G17600	AT1G12290	AT1G17610	AT1G57630	AT3G14470	AT1G61180	AT5G38350	AT3G14460
(SOC3)		(CHS1)	(RPP1-WsB)	(RPP13)	(UNI)		
AT1G64070	AT1G33560	AT1G17615	AT2G32140	AT3G46710		AT5G61240	AT4G33300
(RML1A)	(ADR1)	(TN2)	(TIR-X)	(RPP13-I)		(Cf-5 like)	
AT2G16870	AT1G63350			AT4G27220			AT5G04720
AT3G25510							AT5G38344
AT4G11170							AT5G45440
AT4G11340							AT5G45510
AT4G16890							
(SNC1)							
AT4G16860							
(RPP4)							
AT4G19520							
AT5G38340							
(MSI17.60)							
AT5G41750							
AT5G45000							

Supplementary Table S2: Unique differentially alternatively spliced (DAS) candidates and their event classifications identified in ETI^{HopA1pss}

Alternative 5' splicing	Alternative 3' splicing	Intron retention	Mutually exclusive exons	Exon skipping
AT2G05520 (GRP3)	AT3G56860 (UBA2A)	AT1G08230 (GAT1)	AT1G47830 (AP2S)	AT1G01240 (Transmembrane Protein)
AT3G19190 (ATG2)	AT1G09140 (SR30)	AT1G09140 (SR30)	AT1G60640 (stress response protein)	AT1G05350 (Ubiquitin-like modifier-activating enzyme 5)
AT3G29290 (EMB2076)	AT3G56860 (UBA2A)	AT1G09195 (Ppx-GppA phosphatase)	AT2G23760 (BLH4)	AT1G07119
AT4G00830 (LIF2)	AT1G65270	AT1G14320 (SAC52)	AT3G28100 (UMAMIT45)	AT1G07170 (PHD finger-like domain-containing protein 5B)
AT5G53850 (DEP1)	AT1G05870	AT1G16460 (RDH2)	AT4G17615 (CBL1)	AT1G28330 (DRM1)
AT4G10430 (TMPIT-LIKE protein)	AT5G23610	AT1G20510 (OPCL1)		AT1G50440 (C3H)
AT4G00060 (MEE44)	AT1G50840 (POLGAMMA2)	AT1G25277		AT1G53280 (DJ1B)
AT2G27490 (COAE)	AT4G02450 (P23-1)	AT1G26440 (UPS5)		AT1G54370 (NHX5)
AT1G63260 (TET10)	AT3G27460 (SGF29A)	AT1G50460 (HKL1)		AT1G55810 (UKL3)
AT4G12990	AT2G16940 (CC1)	AT1G61150 (GID8)		AT1G61150
AT4G34265	AT5G64470 (TBL12)	AT1G65270		AT1G69410 (eIF5A-2)
AT1G80245 (Spc97 / Spc98 family of spindle pole body)	AT5G01650	AT1G69530 (EXPA1)		AT1G75560
AT5G54430 (PHOS32)	AT3G47550 (RING/FYVE/PHD zinc finger superfamily protein)	AT1G70620 (Cyclin-like protein)		AT2G06925 (PLA2-alpha)
AT2G25850 (PAPS2)	AT4G00560	AT1G80245 (spec97/spec98 spindle body component)		AT2G16940 (CC1)
AT5G48610 (Myc-like protein X)	AT3G27460 (SGF29A)	AT2G05440 (GRP9)		AT2G20180 (PIL5)
AT1G09195 (Ppx-GppA phosphatase)	AT1G72640	AT2G05520 (GRP3)		AT2G23985
AT4G25880 (APUM6)	AT2G28290 (SYD)	AT2G06005 (FIP1)		AT2G28550 (RAP2.7)
AT5G01260 (Carbohydrate-binding-like fold)	AT5G61310	AT2G32150		AT2G30695
AT3G59640 (PSS1)	AT2G31580 (ICA1)	AT2G32210 (ATHCYSTM6)		AT2G31580 (ICA1)
AT3G57050 (CBL)	AT1G53490 (HEI10)	AT2G32500		AT2G46030 (UBC6)
AT1G70490 (ARF2-A)	AT2G30170 (PBCP)	AT2G36680 (AtVPS37-2)		AT3G02600 (LPP3)
AT2G14260 (PIP)	AT2G05520 (GRP3)	AT2G39950		AT3G11880
AT2G05520 (GRP3)	AT2G32710 (KRP4)	AT2G45660 (AGL20)		AT3G28080 (UMAMIT47)
AT4G19420 (PAE8)	AT3G56860 (UBA2A)	AT2G46225 (ABIL1)		AT3G28956 (RPB4)
AT3G11880	AT2G46225 (ABIL1)	AT3G01500 (CA1)		AT3G61370 (pre-mRNA-splicing factor CWC22-like protein (DUF3245))
AT1G08230 (GAT1)	AT3G51750 (Hypothetical Protein)	AT3G09180 (MED3)		AT4G00060 (MEE44)
AT3G08710 (TRX9)	AT4G12990 (Transmembrane Protein)	AT3G16520 (UGT88A1)		AT4G00560 (NAD(P)-binding Rossmann-fold)
AT1G55810 (UKL3)	AT1G52500 (MMH-1)	AT3G19150 (KRP6)		AT4G02005
AT4G32330 (WDL5)	AT3G21755	AT3G21755		AT4G10430 (TMPIT-LIKE protein)

AT1G70490 (ARF2-A)	AT3G26910 (Hydroxyproline Rich glycoprotein)	AT3G26510 (Octicosapeptide /Phox/Bem1p)	AT4G12990 (Transmembrane Protein)
AT3G23090 (WDL3)	AT1G50000	AT3G29575 (AFP3)	AT4G25050 (ACP4)
AT3G55890 (Protein yippee-like)	AT5G55790 (Hypothetical Protein)	AT3G49160 (Pyruvate kinase)	AT4G34265 (Hypothetical protein)
AT2G36060 (MMZ3)	AT3G60510	AT3G59310 (Solute carrier family 35 protein (DUF914)	AT5G01650 (Tautomerase/MIF superfamily protein)
AT1G61010 (CPSF73-I)	AT5G59210 (Myosin heavy Chain like protein)	AT4G01915 (Hypothetical Protein)	AT5G10350 (RRM)
AT2G28550 (RAP2.7)	AT5G45350 (GPRP-2)	AT4G14385 (Histone acetyltransferase subunit NuA4-domain protein)	AT5G19000 (BPM1)
AT5G51620 (UPF0172)	AT5G11380 (DXPS3)	AT4G16890 (SNC1)	AT5G20165 (kish)
AT2G39730 (RCA)	AT3G62150 (ABCB21)	AT4G20260 (PCAP1)	AT5G26680 (SAV6)
AT4G11970 (YTH family protein)	AT2G38660 (Amino acid Dehydrogenase Family protein)	AT4G21580 (Oxidoreductase, zinc-binding dehydrogenase family protein)	AT5G50950 (FUM2)
AT3G45050 (Transmembrane protein)	AT1G44780 (Translational Initiation factor)	AT4G21585 (ENDO4)	AT5G53850 (DEP1)
AT4G14385 (Histone acetyltransferase subunit NuA4-domain protein)	AT5G24155 (FAD/NAD(P)-binding oxidoreductase)	AT4G23470 (PLAC8)	AT5G56610 (PTPMT2)
AT1G01240 (Transmembrane Protein)	AT3G11880 (DUF2359)	AT4G24580 (REN1)	AT5G62760 (P-loop containing nucleoside triphosphate hydrolases)
AT2G23985 (Hypothetical protein)	AT5G09250 (KIWI)	AT4G33940 (Ring/U-box superfamily protein)	AT5G64500 (Major facilitor superfamily protein)
AT1G07700 (HHL1)	AT1G44770 (AUL1)	AT4G34390 (XLG2)	
	AT1G75310	AT4G35770 (SEN1)	
	AT2G28550 (RAP2.7)	AT4G36980 (CLK4-associating serine/arginine-rich protein)	
	AT2G35510 (SRO1)	AT4G39870 (OXR1)	
	AT4G00355 (ATI2)	AT5G09250 (KIWI)	
	AT2G32160 (SAM)	AT5G11030 (ALF4)	
	AT4G23470 (PLAC8)	AT5G24210 (alpha/beta-Hydrolase’s superfamily protein)	
	AT2G31865 (PARG2)	AT5G27410 (D-aminoacid aminotransferase-like PLP-dependent enzymes)	
	AT1G54217 (Ribosomal protein L18ae)	AT5G45472	
	AT2G44750 (TPK2)	AT5G51620 (UPF0172)	
	AT4G21660 (Proline-rich spliceosome-associated (PSP))	AT5G57120 (MUL3.6)	
	AT1G02840 (SR34)	AT5G58200 (MCK7.7)	
	AT5G49470 (RAF10)	AT5G58720 (PIPC)	
	AT3G22950 (ARFC1)	AT5G66210 (CPK28)	
	AT4G17640 (CKB2)		
	AT1G02120 (VAD1)		
	AT4G21400 (CRK28)		
	AT4G21090 (MFDX2)		
	AT4G21400 (CRK28)		

AT1G70620
AT1G05785 (GOT1)
AT3G26720 (Glycosyl hydrolase family 38)
AT4G28370 (FLY1) E3 ubiquitin ligase
AT4G20380 (LSD1)
AT1G61150 (LisH and RanBPM domains containing protein)
AT2G05520 (GRP-3)
AT4G11900 (S-locus lectin protein kinase)
AT1G13450 (GT-1)
AT2G04700 (FTRB)
AT1G43170 (RP1)

Supplementary Table S3: List of *A. thaliana* proteins enriched with HopA1$_{pss}$ (provided as a separate Excel file).

Supplementary Table S4: List of *N. benthamiana* proteins enriched with HopA1$_{pss}$ (provided as a separate Excel file).

Supplementary Table S5: Supplementary Table 3: Common HopA1$_{pss}$-interacting/co-eluted proteins identified by mass spectrometry in *N. benthamiana* and *A. thaliana.*

	**Identified protein**
**BAIT**	**HopA1$_{pss}$**
1	Decapping protein 5
2	Glycine-rich RNA-binding protein 8
3	THO complex subunit 4B (ALY2)
4	Short-chain dehydrogenase/reductase SDRA
5	DEAD-box ATP-dependent RNA helicase 3, chloroplastic
6	40S ribosomal protein S15-1;40S ribosomal protein S15-3
7	Probable voltage-gated potassium channel subunit beta
8	Bifunctional dTDP-4-dehydrorhamnose 3,5-epimerase
9	40S ribosomal protein S7-3
10	DEAD-box ATP-dependent RNA helicase 52
11	40S ribosomal protein S6-2
12	Polyadenylate-binding protein 4 (PAB4)
13	3-oxoacyl-[acyl-carrier-protein] reductase, chloroplast
14	40S ribosomal protein S19-3
15	60S ribosomal protein L7-2
16	60S ribosomal protein L6-1
17	Trifunctional UDP-glucose 4,6-dehydratase
18	La protein 1 (LA1)
19	40S ribosomal protein S15-4
20	Glutathione S-transferase F10

21	Glycine-rich RNA-binding protein 7

Supplementary Table S6: Structural comparison of HopA1s with PDB entries. (A) HopA1$_{pss}$ (PDB code 4rsw), or (B) HopA1$_{pst}$ (PDB code 4rsx) structures were compared to PDB entries using DALI server (http://ekhidna2.biocenter.helsinki.fi/dali). Top 6 hits from each are shown.

HopA1pss (PDB code-4RSW)

No	Chain	Z	rmsd	lali	nres	% id	PDB description
1	4rsw-A	47.0	0.0	255	255	100	HopA1
2	4rsw-B	44.9	0.3	255	255	100	HopA1
3	4rsx-A	34.8	1.8	251	257	51	TYPE III EFFECTOR HopA1
4	5nvm-A	8.9	3.7	117	176	11	EUKARYOTIC TRANSLATION INITIATION FACTOR 4E TYPE
5	5t46-C	8.8	3.4	117	177	12	EUKARYOTIC TRANSLATION INITIATION FACTOR 4E
6	5nvm-C	8.8	3.2	114	157	10	EUKARYOTIC TRANSLATION INITIATION FACTOR 4E TYPE

HopA1pst (PDB code-4RSX)

No	Chain	Z	rmsd	lali	nres	% id	PDB description
1	4rsx-A	46.4	0.0	257	257	100	TYPE III EFFECTOR HopA1
2	4rsw-B	35.0	1.7	251	255	51	HopA1
3	4rsw-A	34.8	1.8	251	255	51	HopA1
4	6o7z-A	8.7	3.2	116	181	6	PUTATIVE EUKARYOTIC TRANSLATION INITIATION FACTOR 4E
5	6o7y-a	8.5	2.8	112	183	5	PUTATIVE EUKARYOTIC TRANSLATION INITIATION FACTOR 4E
6	6o80-A	8.3	3.0	113	179	4	PUTATIVE EUKARYOTIC TRANSLATION INITIATION FACTOR 4E TYPE

Supplementary Table S7: List of oligonucleotide primers used in this study.

Primer Name	Primer sequence (5'-3')
Stg *Xho*I-*Xba*I-Myc	TCGAGCCCGGGACTAGTATGGAACAAAAACTTATTTCTGAAGAAGATCTGTGAT
ComStg	
*Xho*I-*Xba*I-Myc	CTAGATCACAGATCTTCTTCAGAAATAAGTTTTTGTTCCATACTAGTCCCGGGC
HopA1$_{pst}$_attb1_F	AAAAAGCAGGCTCAATGAACCCCATTCAGTCA
HopA1$_{pst}$_attb2_R	AGAAAGCTGGGTATCATTTCGTGTTTCGAA
*Xho*I-HopA1$_{pss}$ For	GGCTCGAGATGAATCCTATCCATGCA
*Spe*I-HopA1$_{pss}$ Rev	GGACTAGTGTTCGCGCCCTGAGCGCCG
*Xho*I-HopA1$_{pst}$ For	GGCTCGAGATGAACCCCATTCAGTCACG
*Spe*I-HopA1$_{pst}$ For	GGACTAGTTTTCGTGTTTCGAAGGGCCG
qNb*PR1*_RT_F	GGTCAACACGGCGAAAACC
qNb*PR1*_RT_R	GCCTTAGCAGCCGTCATGA
qNb*ACTIN*_RT_F	AACTGATGAAGATACTCACA
qNb*ACTIN*_RT_R	CAGGATACGGGGAGCTAAT
PR5 Tobacco_RT_ F	CCGAGGTAATTGTGAGACTGGAG
PR5 Tobacco_RT_R	CCTGATTGGGTTGATTAAGTGCA

EF1 Tobacco_RT_ F	TGTGATGTTTTTGTTCGGTCTTTAA
EF1 Tobacco_RT_ R	TCAAAAGAAAATGCAGACAGACTCA
HopA1$_{pss}$_RT_F	TGAGCGGTATCCCTCCTGAA
HopA1$_{pss}$-RT_R	CTTTCTGCCAGCGCCATTTT
*MON1*_RT_F	AACTCTATGCAGCATTTGATCCACT
*MON1*_RT_R	TGATTGCATATCTTTATCGCCATC
*PR1*_RT_F	GTGGGTTAGCGAGAAGGCTA
*PR1*_RT_R	ACCTTGGCACATCCGAGTCT
*ICS1*_RT_F	TACTAACCAGTCCGAAAGACG
*ICS1*_RT_R	GAGGCTTGACAACAACTCTGT
*PAD4*_RT_F	TCTTAGCCGAGCCACTCGAC
*PAD4*_RT_R	TTCTCGCCTCATCCAACCAC
*FRK1*_RT_F	CGGTCAGATTTCAACAGTTGTC
*FRK1*_RT_R	AATAGCAGGTTGGCCTGTAATC
*FOX1*_RT_F	GGCTGCACTTCAACCCTTAC
*FOX1*_RT_R	TTACTCTCTGTGGCGTTTGG
*WRKY22*_RT_F	TCCTTCGGAGAGATTCGAGA
*WRKY22*_RT_R	CTGCTGCTACATGGCACACT
*WRKY29*_RT_F	CCCGGAGAAATTCACCATAA
*WRKY29*_RT_R	ATCAGCGGATGGGATCATAG
*WAK2*_RT_F	CGTGTGAGTACACAAATCATCG
*WAK2*_RT_R	TGGTTTAACCTCCTTTGTCTTC
*CYP81F2*_RT_F	AAATGGAGAGAGCAACACAATG
*CYP81F2*_RT_F	ATCGCCCATTCCAATGTTAC
*FLS2*_RT_F	ACTCTCCTCCAGGGGCTAAGGAT
*FLS2*_qPCR_R	AGCTAACAGCTCTCCAGGGATGG
*Luciferase*_RT_F	GACCAACGCCTTGATTGACA
*Luciferase*_RT_R	CGTGCTCCAAAACAACAACGSS
Rabbit β-*globin*_RT_F	TTTGCTAAGCTGAGTGAACTGC
Rabbit β-*globin*_RT_R	CCAGCCACCACCACCTTCTGATA
